# Spatial Fingerprints of Community Structure in Human Interaction Network for an Extensive Set of Large-Scale Regions

**DOI:** 10.1371/journal.pone.0126713

**Published:** 2015-05-20

**Authors:** Zsófia Kallus, Norbert Barankai, János Szüle, Gábor Vattay

**Affiliations:** Department of Physics of Complex Systems, Faculty of Science, Eötvös Loránd University, Budapest, Hungary; University of Gävle, SWEDEN

## Abstract

Human interaction networks inferred from country-wide telephone activity recordings were recently used to redraw political maps by projecting their topological partitions into geographical space. The results showed remarkable spatial cohesiveness of the network communities and a significant overlap between the redrawn and the administrative borders. Here we present a similar analysis based on one of the most popular online social networks represented by the ties between more than 5.8 million of its geo-located users. The worldwide coverage of their measured activity allowed us to analyze the large-scale regional subgraphs of entire continents and an extensive set of examples for single countries. We present results for North and South America, Europe and Asia. In our analysis we used the well-established method of modularity clustering after an aggregation of the individual links into a weighted graph connecting equal-area geographical pixels. Our results show fingerprints of both of the opposing forces of dividing local conflicts and of uniting cross-cultural trends of globalization.

## Introduction

It is almost a truism that the *End of History*, even if it exists, appears to be much farther away from our present than it did at the beginning of the 1990’s [1]. After the falling of the Soviet Union the optimistic atmosphere, shared by many scholars, has been immediately challenged by local conflicts, like the *Yugoslav Wars*. With on-going geopolitical crises the concept of the *Clash of Civilizations* [2] or other less peaceful scenarios became more realistic than ever before in the past of the *post-Cold War* era.

Simultaneously, globalization tries to—in a restricted sense—unify the economic world. Understandably, by the way economy affects the life of people, globalization is not only an economic phenomenon, but it has serious political and cultural aspects too [3], [4]. Global cultural trends and news propagate through the various channels of international communication and the global market of mass media. One of the main platforms of globalized communication is the Internet. On it, beside the usual communication of opinion formers and news agencies, a new, more direct channel appeared by the rise of social networks like Facebook or Twitter. It is a good question if these networks, by getting their users closer to each other, can ease regional conflicts or not. On the other hand, communities formed around specific topics, personalities or interests, e.g. the followers of leaders (like the Pope, the Dalai Lama or various personalities of pop culture or politics) are actively participating in the conversations on a daily basis.

This can be exploited in the following way. We know that the system of interacting people is governed by economic, political and social forces. It is far from its equilibrium state and its evolution is constrained by many factors. By contrast, the communities formed in the free environment of an online social platform can be regarded as ones that represent an inherent partitioning of the system of its users—i.e. one built from the ‘bottom up’ and is able to change quickly. Thus it is interesting to study the self-formed communities of *cybercitizens* that can be projected into the geographical space. Their revealed patterns and boundaries as compared to the administrative borders can highlight friction or connectedness beyond the present geo-political organization of our world.

Recently in [5] and [6] the authors analyzed telephone call activity data in order to answer the following question: *“Do regional boundaries defined by governments respect the more natural ways that people interact across space?”* Their method consisted of the inference of a spatially embedded aggregated network and the partitioning of the graph based solely on topological information. Surprisingly, the clusters showed compact forms in space that were in high agreement with the administrative boundaries of *Great Britain*, *France*, *Belgium*, *Italy*, *Portugal*, *Ivory Coast* and *Saudi Arabia*. While such rich data set can reflect the connectedness by telephone calls at a high level of accuracy through the measurement of everyday communication between people it is not without limitations. First, typically coming from a single operator of communication services, despite a high spatial resolution the geographic coverage is limited to small or medium scale territories, such as cities or single countries. Another disadvantage of such databases is their inaccessibility for the general research community. Usually they are not freely available and their use is restricted by varying privacy regulations.

This is why, up until now, it was not possible to carry out such analysis on large-scale geographic regions. Contrary to previous studies usually presenting results on the level of cities or single countries, the above mentioned questions of cultural and political conflicts and opposing globalizing interests require the collection and study of social ties with extensive geographic coverage.

As emphasized in [7], the newly accessible large-scale data on human interactions, e.g. the digital footprint of active Internet users, opened an entirely new window for those who are interested in such questions.

The aim of this paper is to present our findings on the spatial structure of an extensive set of large and medium scale regional communication networks. More specifically we show how the social ties formed on the online platform of Twitter are reflected in the spatial projection of the clustering of its regional subgraphs covering four continents. In order to accurately quantify the relative strength of social connections we collected messages of millions of interacting public accounts with accessible geographic information over an extended period of time. We used the gathered data set to create a list of active users of known locations and uncovered their spatially embedded graph. Then we performed modularity clustering on its different regional representations.

Our results show examples where geo-political borders or spatial proximity are surprisingly good predictors in the projected topological cluster formation. On the other hand there are regions with spatially scattered clusters. In this latter case, ties strengthened by long-range cultural and economical factors are the predominant ones.

## Materials and Methods

### Creating spatially embedded regional graphs

In order to measure the strength of social ties between people of different locations, we exploit the rapidly growing activity on online social networks reinforced by the rising popularity of smart devices.

As detailed in [8] Twitter provides a freely accessible “sprinkler” stream, and we used it over a 16 months period (between January of 2012 and May of 2013) to create a large database of more than three and a half billion short messages, i.e. *tweets*. From this collection a list of active geo-users with public accounts was created. Using messages with geographic information we were able to determine an average location for the owner of an account that corresponds to the center of the largest spatial group of locations of that user.

To connect these users we can choose from different link definitions. For this work we chose to only link two users if a mutual communication channel is present between them. This is intuitively a more stable definition than e.g. the more volatile directed message counts that would be inferred from the restricted sample messages. Twitter users can *follow* any other public account and once this channel is open, they receive an automatic notification of their new tweets. Based on the assumption that mutual links are more likely to connect users who are related in the “real world” as well, we only keep bidirectional follower relations reflecting mutual interest between the users.

We identified over 16 million regularly used accounts with public geographic information. From those we selected the 6 million most active ones and collected their follower links. This collection of follower graph links was performed in a separate discovery campaign. Its speed was only limited by the public Twitter API rate limits.

As a result we created the spatially embedded, undirected graph of more than 5.8 million users. We note that although not the entire set of accounts is used by everyday people, for this specific analysis, this is a strength of the data set, since economically motivated companies as well as public figures use the platform to reach out and to stay in contact with their respective target audiences.

To incorporate geographical adjacency relation into this graph, we aggregated the user-level links into non-overlapping geographical cells. We used the method of HEALPix, originally developed by NASA JPL for astrophysical purposes [9], to form equal-area pixels on the surface of the Earth. A HEALPix pixelization is described by a single parameter, *N*_*s*_. If *N*_*s*_ is given, the boundaries and the shape of the the spherical lattice of cells are absolutely determined and the number of pixels is $12N_{s}^{2}$. We set *N*_*s*_ = 650 which gives a pixel area of approximately 100 km^2^. The output of the HEALPix method is the equal-area grid. (This is also visible on the map figures where we kept the pixels with non-zero user number and colored them according to the clustering results.)

We start the closer examination of a larger geopolitical region by identifying the induced subgraph of the users located to this region. Next, we aggregate the users into local groups corresponding to the covering of the geographical region by HEALPix pixels. This step can be viewed as a pre-clustering and results in a partition of the original network. After the isolation of the giant component of the partition, it served as the input of the modularity optimization algorithm in order to find its non-overlapping communities. Detailed information on the connectivity matrices can be found in Table 1 and Table 2. Histograms of the distribution of number of users per pixel are given in SI (Figures C-E in S1 File).

**Table 1:** Regional graphs.

Region	Users	Edges	Pixels
*Canada*	91,813	447,541	3,778
*US*	1,622,152	17,076,244	31,542
*South America*	625,976	6,184,291	10,975
*EU*	1,162,630	10,566,764	22,904
*Europe*	1,545,567	14,196,996	30,644
*Germany and Turkey*	252,167	2,610,222	4,456
*Former Yugoslavia*	9,274	97,184	724
*UK*	411,427	3,659,786	2,141
*Spain*	304,369	2,778,297	4,126
*France*	79,194	584,040	3,490
*Germany*	23,991	147,301	2,117
*SE Asia, China and Japan*	961,669	8,039,210	13,065
*Japan*	185,306	1,282,672	2,995
*India*	18,370	61,739	1,529

Detailed information on the size of the regional subgraphs.

**Table 2:** Regional graph partitions.

Region	Pixels in the g. c.	Edges in the g. c.	Clusters	*Q*_*found*_
*Canada*	3,747	447,517	8	0.58
*US*	31,532	17,076,236	15	0.46
*South America*	10,963	6,184,282	12	0.68
*EU*	22,888	10,566,750	10	0.67
*Europe*	30,617	14,196,973	11	0.73
*Germany and Turkey*	4,444	2,610,211	11	0.3
*Former Yugoslavia*	724	97,184	7	0.52
**UK*_*N*_*s*_ = 650_*	2,404	3,659,805	14	0.37
*Spain*	4,126	2,778,297	16	0.54
*France*	3,488	584,038	9	0.3
*Germany*	2,105	147,292	10	0.26
*SE Asia, China and Japan*	13,033	8,039,187.5	13	0.7
*Japan*	2,995	1,282,671	10	0.19
*India*	1,485	61,709	8	0.36

Detailed information on the size of the giant component of the regional subgraphs and the found partitions.

### Clustering method

Our clustering method relies on the notion of modularity [10]. If a partition of the nodes of a network is given, the modularity tries to quantify the possibility that the partition is formed by a set of relatively dense clusters that are only weekly connected to each other. The higher the modularity of a partition, the more probable that it contains heavily linked communities with rare occurrence of edges between them. One disadvantage of the concept of modularity is the computational intractability of finding the partition with the highest measure. From the beginning of the popularization of the concept, various heuristic methods have been proposed [11]. One of the most successful among them is the random greedy algorithm, introduced in [12]. In one step, the algorithm chooses some topologically adjacent clusters in a random way and selects those two whose merging can increase the modularity the most or, if this is not possible, decrease it the least. By performing independent simulations on the same input graph repeatedly we can get better and better approximations of the global optimization problem. We adjusted our search parameters to the size of the input graphs, and performed a simulation with higher number of repetition for regions of size exceeding a threshold. Specifically, when the number of nodes of an input graph exceeded 10,000 the simulation was run 3,000 times, and only 1,000 times for the smaller ones. This limiting trade-off between higher repetition number and size of the graph is the reason of restricting this study to the regional graphs, and not analyzing the entire user-level follower graph. As a new research direction this possibility is mentioned in Sec. *Conclusion and Outlook*. A short description of the definition of modularity and the random greedy modularity clustering method can be found in SI (S1 File).

An advantage of this method is its tendency to find near optimal partitions with higher cluster sizes. This can allow us to find medium-sized clusters in large-scale regional graphs, and small clusters in medium-sized graphs. On the other hand, this also can have the effect of finding a clustering where the size of largest cluster is disproportionately large. In order to diffuse this covering effect, we performed a second level clustering on the subgraph of such clusters, and presented the revealed sub-structure as part of the first order results.

It is important to emphasize that our goal and method is fundamentally different from those associated to the notion of spatial clustering. In fact, we only use the spatial information when pixels are created *before* the main clustering process, and when the obtained partition is projected to the map—i.e. *after* the clustering process. Only the abstract topological relations are used by the clustering algorithm itself.

To measure the effect of spatial density on the community structure we performed the following analysis. We create a random rewiring, i.e. a new user-level graph, by swapping the links between the users randomly. The total number of swaps performed is ten times the number of the user-level links. By keeping the degrees and positions of the users the same as in the original graph, the impact of the spatial density on the clustering algorithm is also the same. Then we create the new geo-pixel graph with new aggregated weights. The clustering algorithm can be run the same way repeatedly, and we choose the community structure with the highest modularity found.

### Quantitative comparison of partitions

Once the clustering simulations were done a comparative study was performed for each regional graph. Our method gave a community structure that aimed at maximizing the modularity value. Another partition was determined according to the administrative borders [13] as follows. Each geo-pixel was assigned to the administrative unit that contained its centroid or if none of the units did, the pixel was assigned to the closest one. The *goodness* of our clustering and of the administrative units was compared by calculating the modularity value for both partitionings. Their *difference* can be also quantified. Several measures were introduced in the literature. We calculated both the *adjusted Rand index* [14] with definition based on a statistical background and the *normalized mutual information* [15] coming from an information theory background. Detailed description of these measures is given in the SI (S1 File).

## Results and Discussion

In this section we present a quantitative evaluation of our results followed by a descriptive summary of the projected regional clusters summarizing each region’s highlights. In SI we include all regional data sets necessary to reproduce our presented projection results (S2 File).

**Fig 1:**
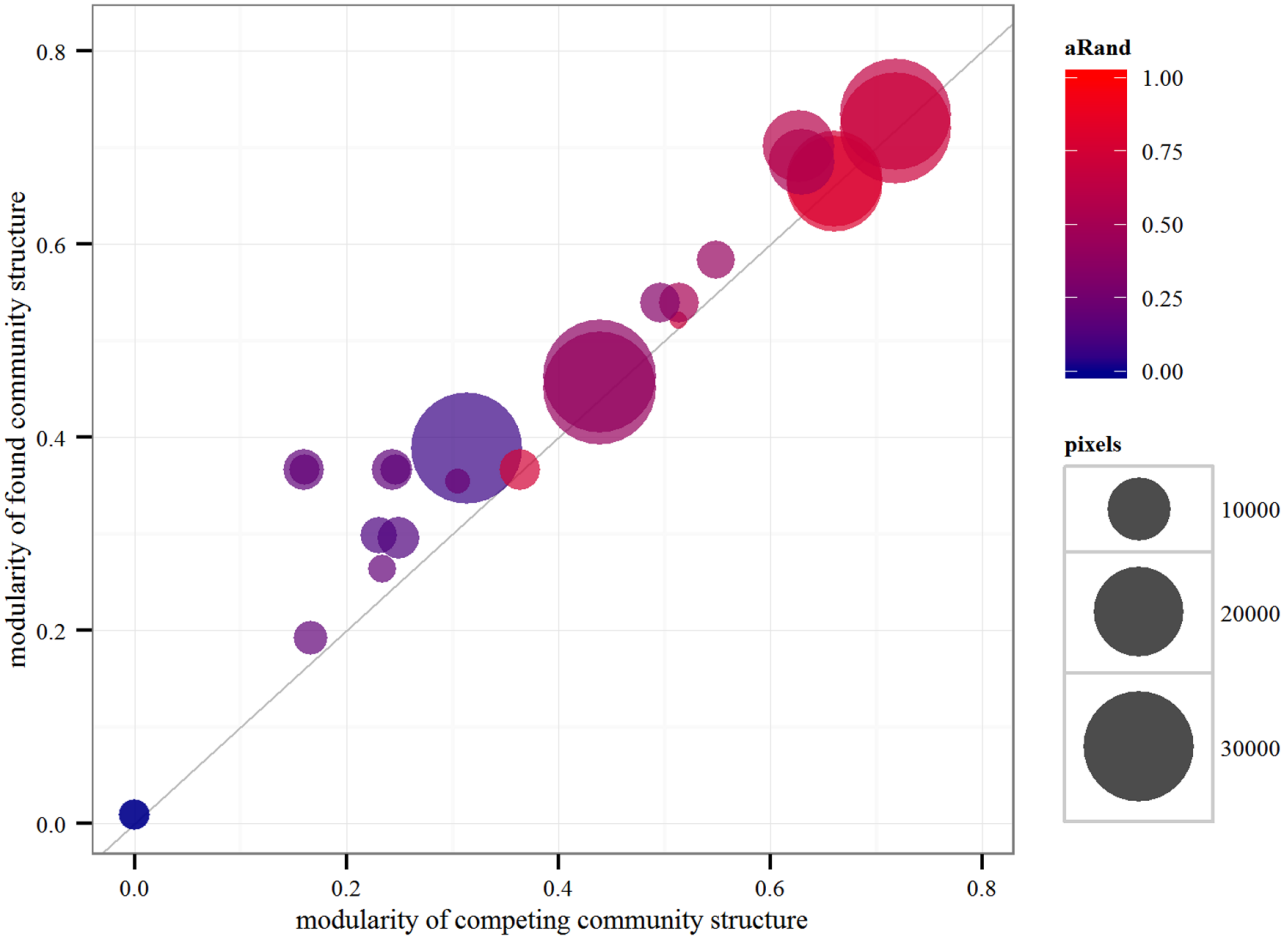
Regional graph partition comparisons. Each point represents a comparison of two partitions of the same regional graph, i.e., one line from Table 3. The modularity of the partition found by our method is shown on the vertical axis while the modularity of the competing partition is given on the horizontal axis. Color shows the difference between the two partitions measured by the *adjusted Rand index* and the area of the disk is proportional to the *number of pixels* in the graph.

A short randomization study illustrates the structure-forming strength of the network connections by the example of the *UK* graph. Another short study illustrates the effect of the chosen HEALPix grid size, also by the example of the *UK* graph. Next, a comparative analysis is presented between the found community structures and the administrative units in the continental and single-country regions illustrated on Fig 1 and summarized in Table 3. Finally, we give a short descriptive summary of highlights of the community structures revealed, giving plausible explanations where we can. A detailed description of the clusters can be found in SI (S1 File).

**Table 3:** Regional graph partition comparisons.

Region	Competing partition	*Q*_*found*_	*Q*_*competing*_	𝓢_n_	ℛ_ad_
*Canada*	*adm1*	0.584	0.549	0.566	0.418
*USA*	*adm1*	0.463	0.439	0.615	0.377
*combined USA*	*adm1*	0.451	0.439	0.632	0.423
*South America*	*adm0*	0.684	0.629	0.725	0.522
*EU*	*adm0*	0.668	0.66	0.742	0.749
*combined EU*	*adm0*	0.663	0.660	0.775	0.804
*Europe*	*adm0*	0.734	0.718	0.708	0.656
*combined Europe*	*adm0*	0.720	0.718	0.742	0.707
*Switzerland in Europe*	*adm1*	0.389	0.313	0.279	0.063
*Germany and Turkey*	*adm1*	0.295	0.249	0.345	0.127
*Former Yugoslavia*	*adm0*	0.521	0.513	0.707	0.711
**UK*_*N*_*s*_ = 650_*	*adm1*	0.366	0.160	0.454	0.227
**UK*_*N*_*s*_ = 650_*	*adm2*	0.366	0.246	0.579	0.172
*Spain*	*adm1*	0.538	0.514	0.680	0.507
*Spain*	*adm2*	0.538	0.497	0.645	0.332
*France*	*adm1*	0.298	0.230	0.264	0.112
*Germany*	*adm1*	0.264	0.234	0.234	0.189
*SE Asia, China and Japan*	*adm0*	0.701	0.627	0.707	0.585
*Japan*	*adm1*	0.192	0.166	0.406	0.184
*India*	*adm1*	0.355	0.304	0.313	0.211
**UK*_*N*_*s*_ = 920_*	**UK*_*N*_*s*_ = 650_*	0.367	0.364	0.735	0.750
**UK*_*N*_*s*_ = 920_*	*adm1*	0.367	0.160	0.435	0.180
**UK*_*N*_*s*_ = 920_*	*adm2*	0.367	0.243	0.56	0.174
*rewired *UK*_*N*_*s*_ = 650_*	*adm1*	0.009	0	0.001	0
*rewired *UK*_*N*_*s*_ = 650_*	*adm2*	0.009	−0.001	0.057	0

Modularities of the found and of the competing partition of the same regional subgraph and their two measures of difference: normalized mutual information and adjusted Rand index. (*combined* refers to result mixed with partial second-level clustering. *adm0* denotes the country-level, while *adm1* and *adm2* the corresponding first- and the second-level subnational administrative divisions, respectively.)

### Quantitative evaluation of regional partitions

#### Effect of randomization on clustering result

**Fig 2:**
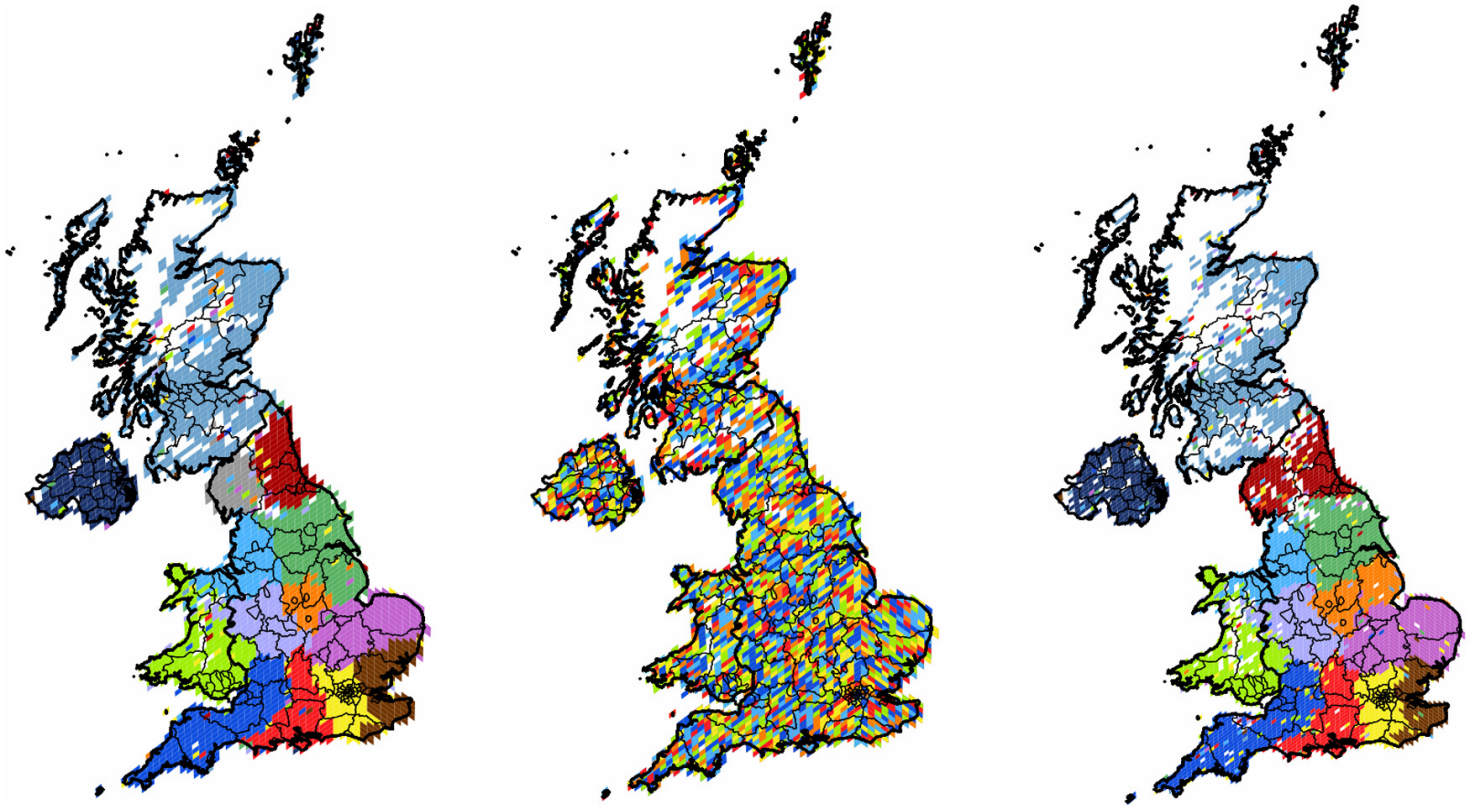
Clustering of the United Kingdom. The first panel shows the 2,404 pixels forming 14 well defined clusters with modularity value of 0.37. The second panel shows the clusters of the randomly rewired graph with 6 clusters. The third panel shows the result of the graph with finer HEALPix grid with 13 clusters. On each map, each cluster of geo-pixels has a different unique color. The boundaries between the four countries are shown in thicker lines, while the second-level subnational division is indicated with finer lines, depicting the second type of administrative units analyzed.

To illustrate how strongly the lack of the topological structure would impact our results, we use the *UK* as an example. We compare results obtained for the original and for the randomized user-level graphs. Running the algorithm described in Sec. *Materials and Methods* for both graphs we see how significantly the goodness of the clustering is changed. While the original case has modularity value of 0.367, the randomized result is two orders of magnitude smaller, only 0.009. In addition we can compare the projected clusters (14 and 6 for original and randomized graphs respectively) and their compactness in the geographic space. The results are shown on the first and second panels of Fig 2. We see how the randomized connections with the same spatial distribution give a completely and evenly scattered embedding. This is in contrast to the real clusters, that are compact and well defined in space. This example shows that the spatial structure of communities revealed after the projection is highly influenced by the topology and is not completely determined by the spatial distribution of the user locations in itself.

#### Effect of grid size on clustering result

To illustrate how the choice of the fixed parameter of the pixel size would affect our results, we use the *UK* graph again. We compare results obtained from the original grid and from a finer one, with 50 km^2^ pixel size, i.e., where the HEALPix parameter is set to *N*_*s*_ = 920. Running the algorithm 3,000 times we found a partition of modularity 0.367 and 13 cohesive clusters. As it can be seen on the third panel of Fig 2 there is a great overlap and significant similarity between the two partitions, as we will detail it in the next section (see Table 3 and Fig 1). The main differences are only the swapping of small cohesive pixel groups between neighboring clusters and a little more satellite presence in general.

#### Comparative study of administrative regions and projected clusters

We compare the found best partition of each geo-pixel graph to the partition of the same graph enforced by the respective administrative borders. We compare their goodness and quantify the difference between these partitions using abstract measures of difference. The goodness of a partition is measured by its modularity value. When the graph being partitioned is the same, higher modularity value always means better quality topological clustering. In addition, we can measure the difference between two partitions using normalized measures. We calculated two well-established measures: the *adjusted Rand-index* ℛ_ad_ and the *normalized mutual information* 𝓢_n_. Detailed description of these measures can be found in SI (S1 File).

Our method is as follows. According to the algorithm presented in Sec. *Materials and Methods*, we select a region and take the giant component of its geo-pixels as our input graph. First we cluster it using the random search method to find close to optimal topological clusters. Then we keep the same graph and take the partition enforced on it by the administrative boundaries: for each pixel we assign the administrative unit that contains (or, in case of external centers, is closer to) its centroid. Once the two partitions are created we calculate the respective modularity values and their differences. We repeated this process for all regions. We used country borders for the continental regions and subnational administrative boundaries for the single-country regions. As an example, for the *UK* region we analyzed an additional partition: the regions of the smaller subnational administrative units in addition to the the larger units of its countries.

Fig 1 illustrates our results that we summarized in Table 3. On this scatter plot each point represents a comparison between our found modularity value (shown on the vertical axis) and the modularity of a competing administrative partition of the same graph (horizontal axis). The size of a disk shows the number of pixels of the input graph and the color shows the difference between the two partitions as measured by the *adjusted Rand index*. (Semi-transparency is used to show overlapping cases). For the *UK* graph we have five additional comparisons listed at the end of Table 3: one between the found results of original graph and of the randomized one, two between the found result of the small grid graph and the first- and second-level subnational administrative units for this graph, and finally two between the found result of the randomized graph and the first- and second-level subnational administrative units for this graph. We note that the included results for the randomized graph of the *UK* are visibly and significantly under-performing all other clusterings.

In general it can be seen that the results found with the random modularity optimization algorithm outperform all other clusterings for all of the regions. Thus the found clusters give better quality partitions. On the other hand, we can also state that the difference between the administrative units and the network communities is not extreme, i.e., there is a significant overlap between the clusters. This is true not only for the single-country networks, but also on the continental scale.

### The projected regional clusters

In this section we give a summary of the clusters found in each of the networks. We see that fingerprints of globalization and of local conflict are present as well. A full length, detailed descriptive study can be found in SI (S1 File), and here we highlight those results that are readily interpretable by plausible explanations, although not always expected to be seen in this data set.

#### General remarks

Our extensive data set of Twitter users worldwide presents a unique opportunity to study regions that reach far beyond the borders of single states or countries. Our limiting factor is the inhomogeneous spread of the use of Twitter network and the fact that the freely available messages represent only a half percent sample of the complete data set. The latter is partially counterbalanced by the aggregation over time (of the messages and hence the number of users and their message points) and space (by starting the clustering in pixels). We note that the last comparative study between the full and the sampled stream of Twitter messages [16, 17] showed that in case of the messages coming from accounts with available geographic coordinates, the sample represents a much higher part that equals to 90.1% of the total of the geotagged messages.

As seen in Table 2 the studied subgraphs can be divided into two categories: large-scale regions covering the area of several states or countries and the subgraphs consisting of a single country. Looking at the results one can see that there is no real correlation between the number of clusters found for each regional graph and the size of the graph measured by the number of pixels.

In all of the cases, discussed in the following sections, we find that the clusters resulting from purely topological information form units that are more or less geographically cohesive. These clusters sometimes redraw the boundaries of the geopolitical regions. They are cohesive in the sense that the majority of their pixels are well connected on the map, usually with only a few *satellite pixels* that reach out to remote areas. This general behavior is only different in the case of the few exceptionally influential, central clusters. These tend to be characterized by an overly expending number of satellite pixels colonizing most of the territories.

#### Community Structures in North America

In this region Twitter is well known across the countries, and the areas covered by our data set closely follow the populated places of the continent. Here we show our results for the two separate subgraphs of *Canada* and the *Continental United States*.

**Fig 3:**
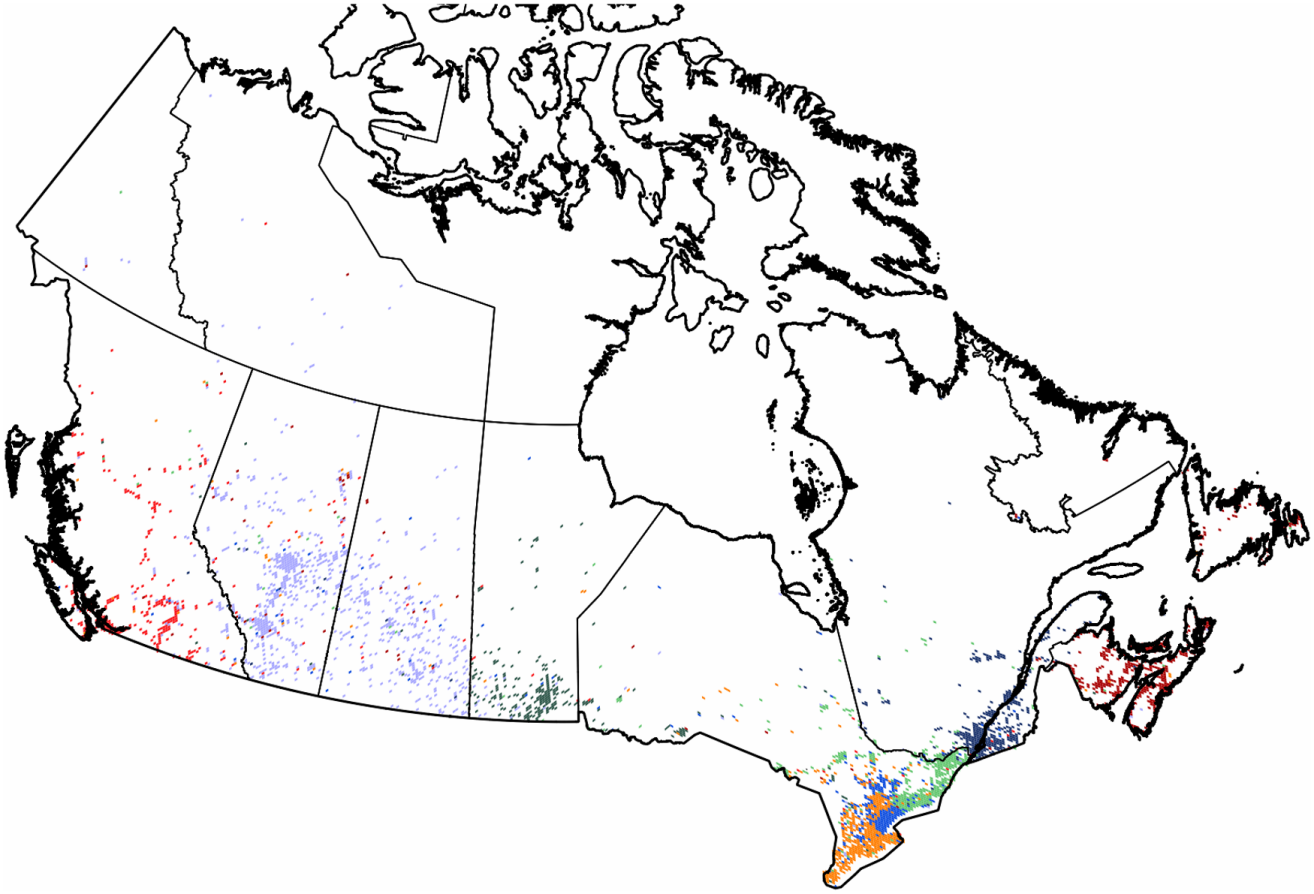
Clustering of the subgraph of Canada. The 3,747 pixels form 8 well defined clusters with modularity value of 0.58. Each cluster of geo-pixels has a different unique color. The state boundaries are marked depicting the administrative units of this graph.

**Canada**: When analyzing the subgraph of *Canada*, as seen in Fig 3, it is well divided into smaller regions. Eight clusters are formed, giving a modularity value of 0.58. Within these clusters we find the well-known French language territories forming clearly separated communities. The northern territories of lower population densities are only sparsely occupied by the Twitter geo-users, the clusters of the southern part follow the administrative boundaries. While *Québec* forms its own cluster only swapping a few satellite pixels with its western neighbor, its center of gravity is divided between the cities of *Montréal* and *Québec*. There is a smaller cluster formed around the city of Toronto.

**Fig 4:**
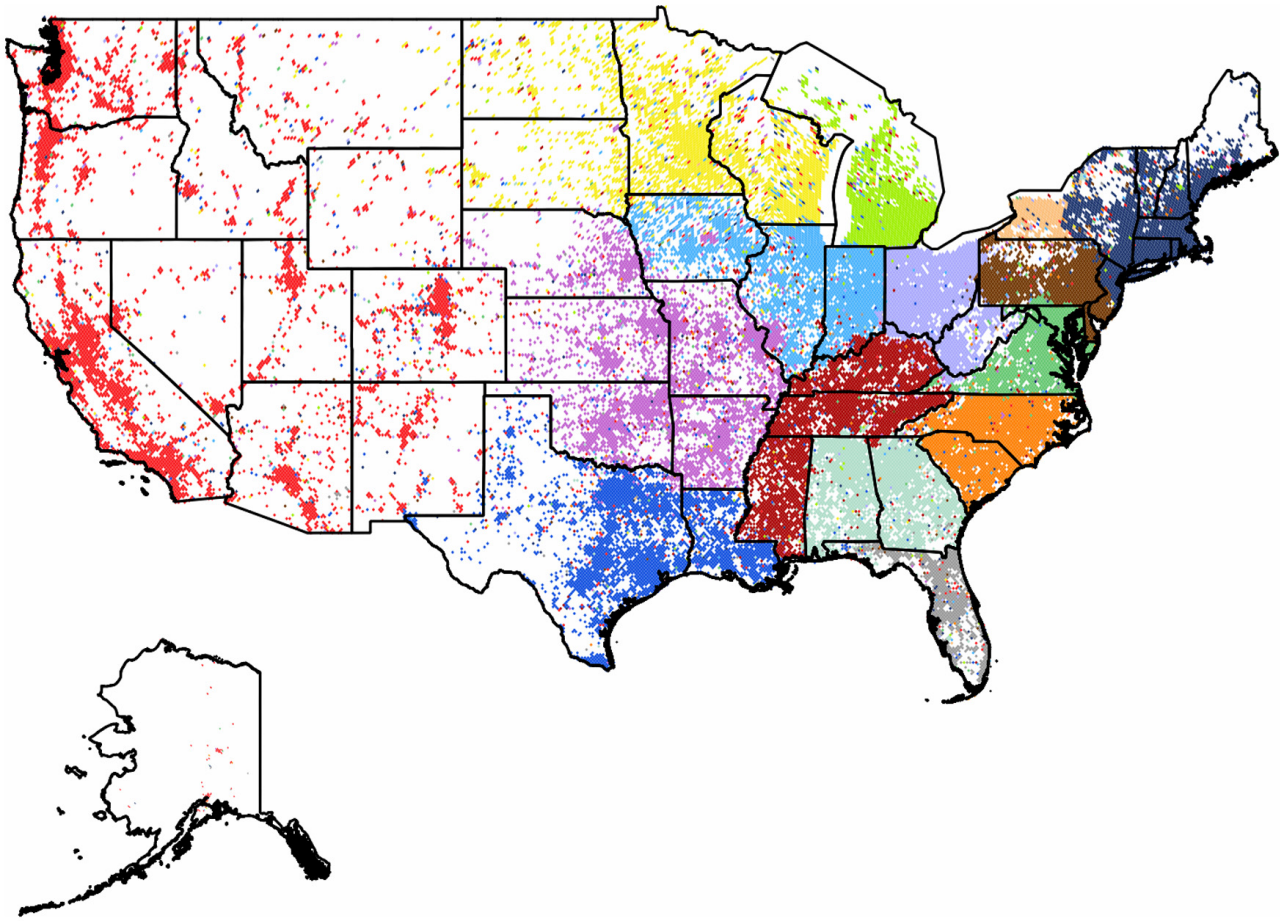
Clustering of the subgraph of the Continental US. The 31,532 pixels form 15 well defined clusters with modularity value of 0.46. Each cluster of geo-pixels has a different unique color. The state boundaries are marked depicting the administrative units of this graph.

**The Continental United States**: As seen in Fig 4 the *US* mainland territories form fifteen well separated clusters with a modularity value of 0.46. The *US* is effectively divided into a western and an eastern part, separated by a sparsely populated central region that also corresponds to the smaller Twitter user counts of this intermediate area.

**Fig 5:**
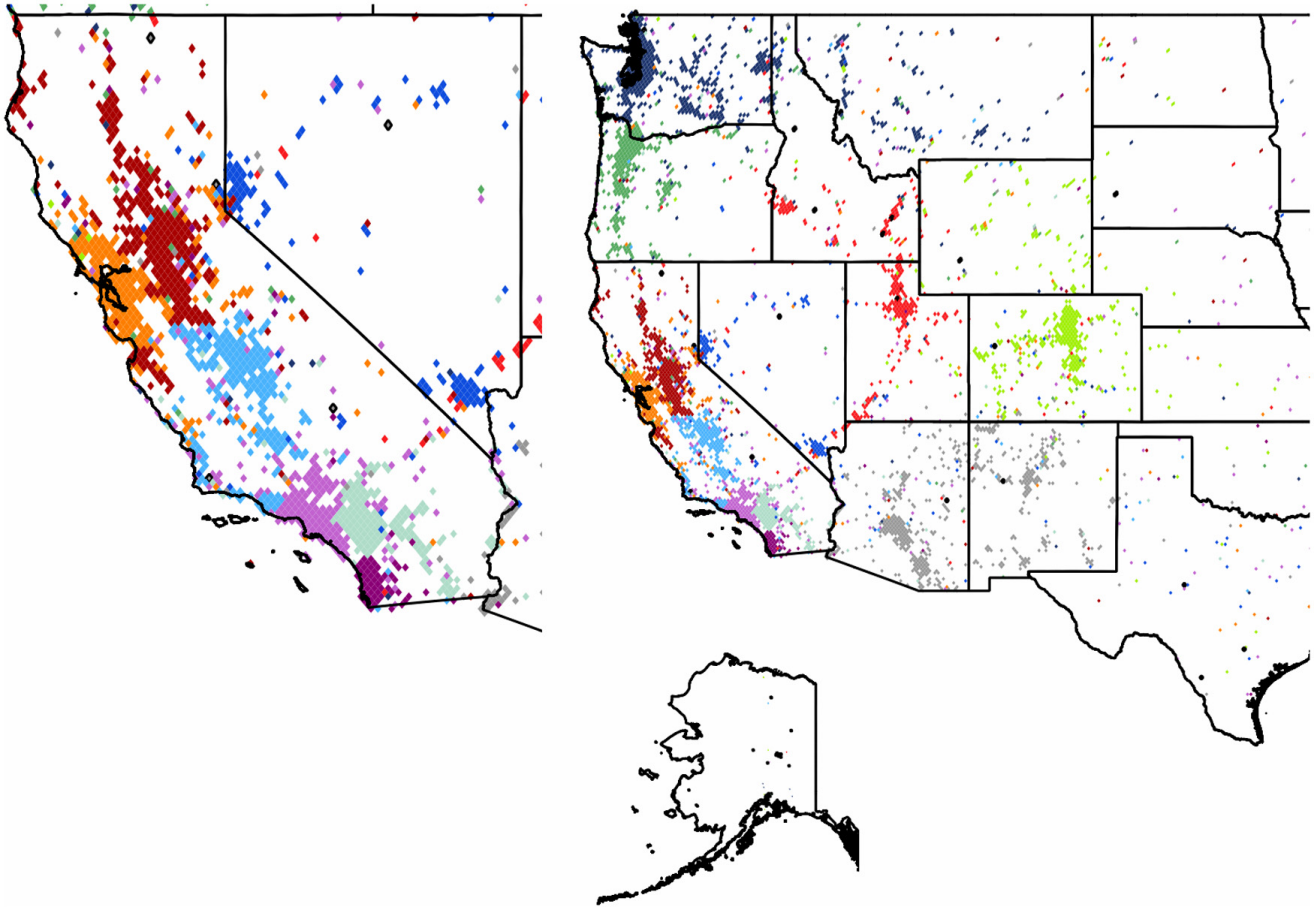
Second level partitioning of the Western US cluster. The 4,507 pixels form 13 well defined subclusters with modularity value of 0.51. The insert shows the coastal communities formed around urban areas. Each second-level cluster of geo-pixels has a different unique color. The state boundaries are marked depicting the administrative units of this graph.

In the eastern part the clustering mostly transpires by the formation of units that follow the administrative borders with high precision and include a single or multiple states. A few states are divided into several clusters. As we approach to the west, the occupied pixels become rather sparse, and form a single cluster. This community penetrates through satellite pixels mostly to the northern and central clusters. Although this spread reaches multiple clusters, it is never forming a well-defined geographically cohesive unit in any of them. For further detail we performed a second-level clustering on this large western community as a subgraph in itself. The result is depicted in Fig 5. The state of *California* is a special case. This is the home of highly active Twitter users, second only to the *UK*. According to our expectations it is formed by several smaller clusters that are highly active on their own. The center of gravity of these clusters correspond to large cities of the state.

#### Community Structure in South America

**Fig 6:**
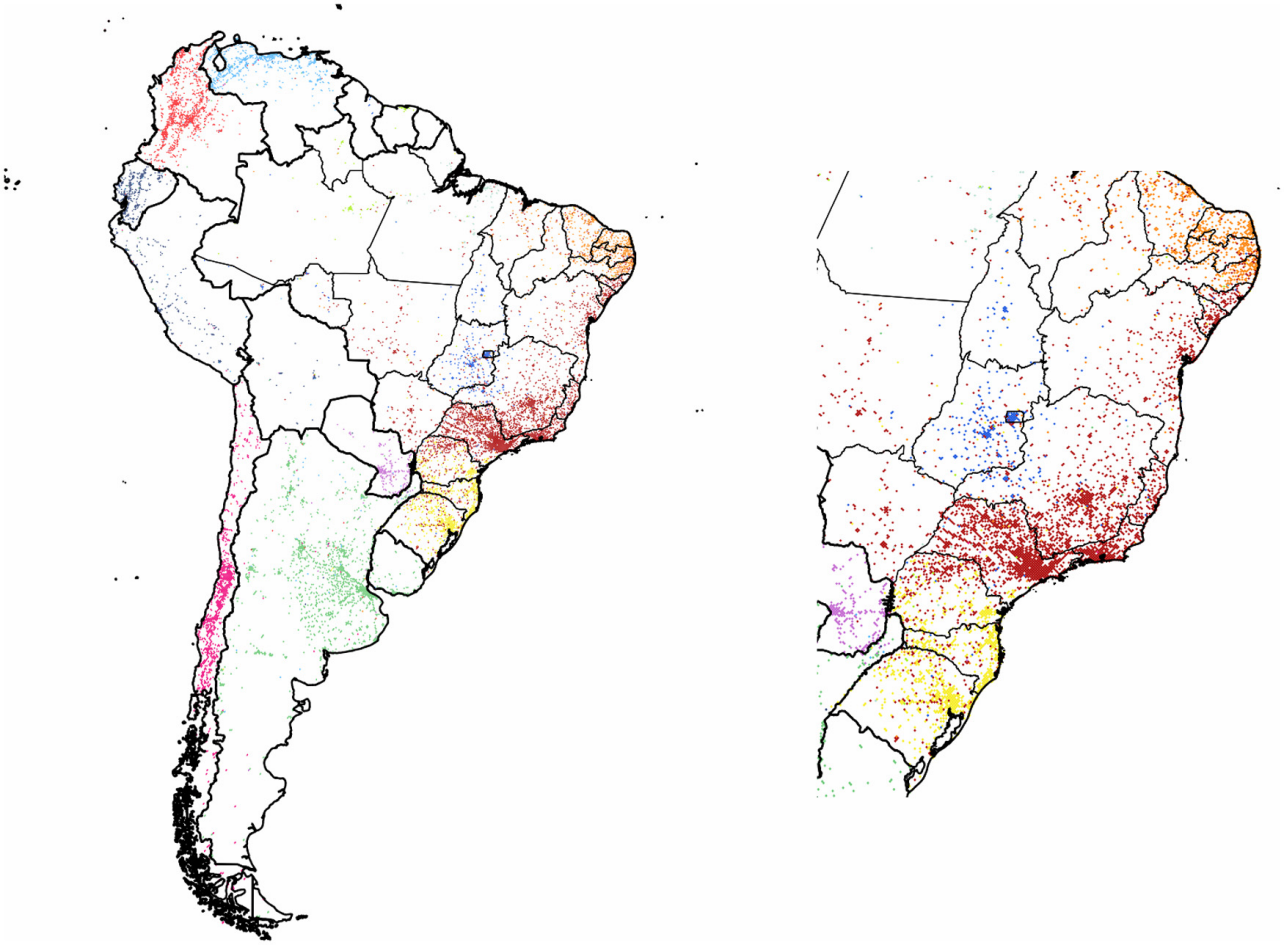
Clustering of the countries of South America. The 10,963 pixels form 12 clusters with modularity value of 0.68. The insert shows the coastal communities. Each cluster of geo-pixels has a different unique color. The country borders are marked with thicker black line depicting the administrative units of this graph. The thinner lines mark the second-level administrative units of Brazil.

We performed a first level clustering on the subgraph of *South America*. As it can be seen in Fig 6, it resulted in a partition of twelve clusters with a high modularity value of 0.68. The discovered communities form two distinct groups: the six clusters cutting up the area of *Brazil* reaching out to the few pixels of *Suriname*, *Guyana* and the overseas department of *French Guiana*—and the rest of the countries. The latter consists of clusters of single or multiple countries. This unbalanced division could be explained by the western part of the continent being Spanish speaking and therefore lacking of any language barriers, or also by *Brazil* being the most active country of the region and big enough in itself to be partitioned into smaller communities around its larger urbanized areas. Although there are clusters with satellites in each, they are not forming a bridge between these separated groups.

#### Community Structures in Europe

**Fig 7:**
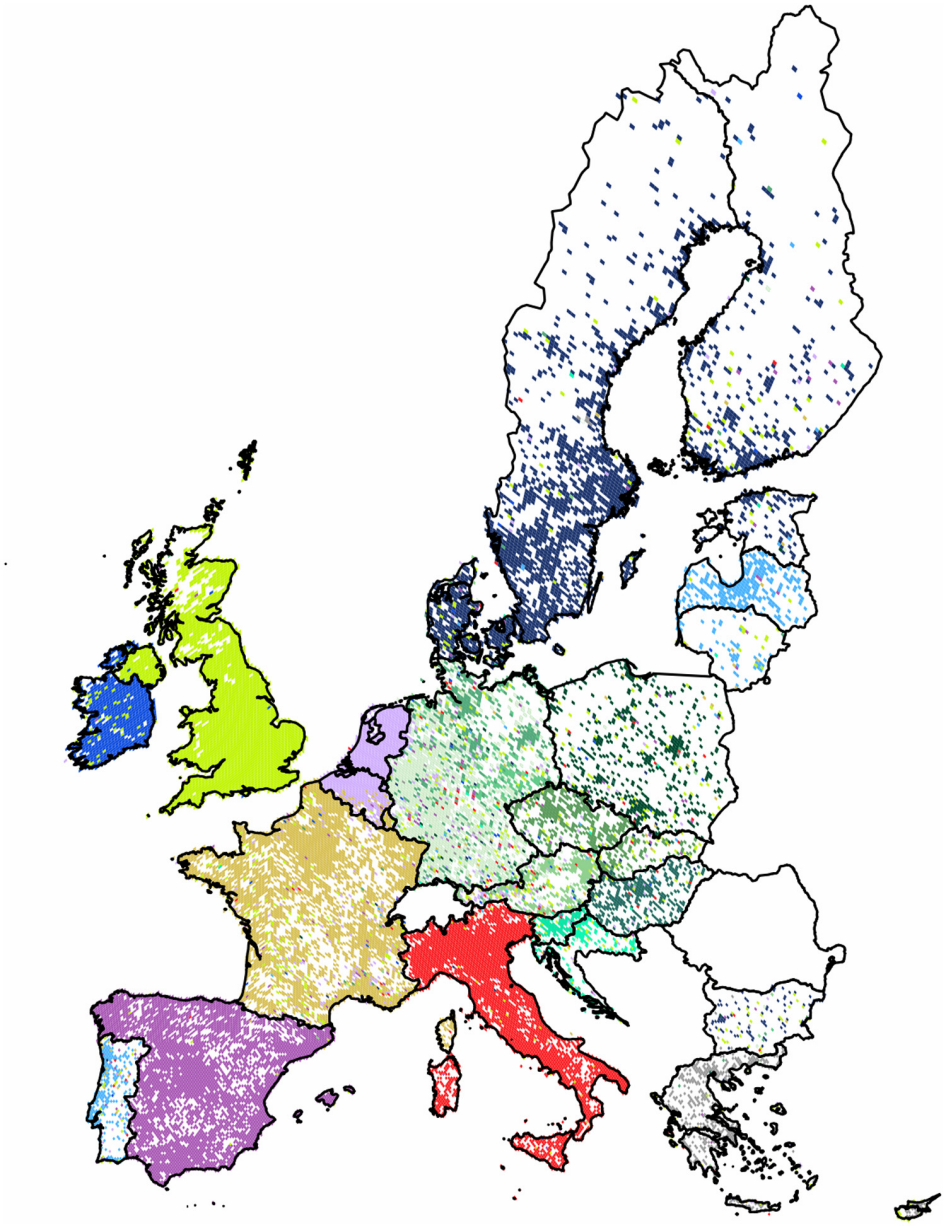
Clustering of the 28 member countries of the European Union combined with second-level results. The 22,888 pixels form 10 well defined clusters with modularity value of 0.67. Here the central cluster is replaced by the 10 sub-communities obtained by its second-level partitioning. Each cluster of geo-pixels has a different unique color. The sub-communities of the central cluster have different shades of green. The borders of countries are marked depicting the administrative units of this graph.

**Fig 8:**
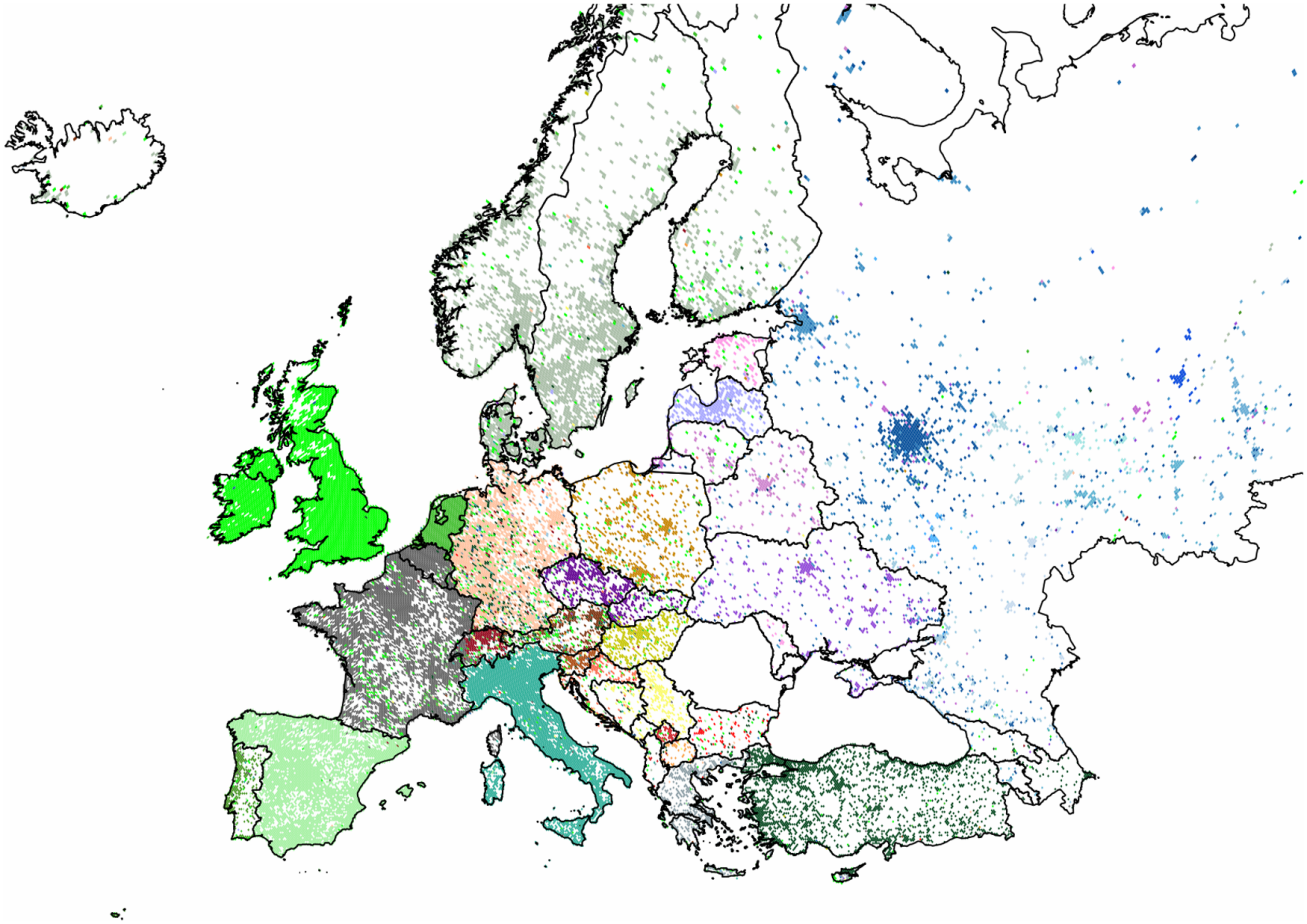
Clustering of the countries of the European continent combined with second-level results. The 30,617 pixels form 11 clusters with modularity value of 0.76. The two largest clusters are replaced by the 11 and 18 sub-communities obtained by their respective second-level partitionings. Each cluster of geo-pixels has a different unique color. The first-level clusters and the sub-communities of the two largest clusters have different shades of green, blue and brown, respectively. The country borders are marked depicting the administrative units of this graph.

First we formed the subgraph of the countries of the European Union. Next we extended our analysis to the communities of the entire continent extending from the *UK* to *Russia*, also including the territories of *Georgia*, *Armenia* and *Azerbaijan* for geographic integrity. Additionally, we show our results for a list of standalone countries. In general, as it can be seen in Figs 7 and 8, the use of Twitter is more dense in the western part of the continent, with the *UK* having one of the most active users worldwide, concentrated around the *London* area. The popularity of Twitter closely follows the urbanized areas, but has significantly lower level of penetration in the *Central* and *Eastern European* regions. Large urbanized areas of *Russia* and of *Turkey* are well represented. We note the apparent disadvantage of this data set that is its lack of any recorded tweets from *Romania* and from *Malta*.

**The European Union**: In this region the partitioning had a high modularity value of 0.67, revealing ten compact clusters with well defined areas and mostly negligible outreach with some exceptions. The *UK* has the most influential cluster, marking its presence with satellites in every country, with least coverage in *Spain*. *Germany* dominates the central area of *Europe*, reaching all the way to the countries of the *Former Yugoslavia*. *Cyprus* forms a cluster with *Greece*. As an interesting detail, we see satellite pixels in *Greece*, mainly coming from western clusters present on the shores of the country of two thousand islands. This phenomenon probably shows the attraction of foreign tourists, while the possible language barrier contracts the central areas to form the separate community of *Greece*. For further detail we performed a second-level clustering for the central subgraph as a single input to the algorithm. The combined map is shown on Fig 7. The result showed ten well defined clusters of a size reaching the lower limit of ten pixels and four outliers.

**The European Continent as a whole**: In this case, we see a very strong partitioning as measured by the high modularity value of 0.73. Out of the eleven clusters there are two with significant outreach of satellite pixels, while the other clusters show geographic cohesiveness. Disproportionately large clusters are formed by *Germany* and by *Russia*. The first reaching all the way through the central region and the latter containing mostly the eastern countries and a few satellite pixels. On these large clusters we performed secondary clustering giving eleven and eighteen new clusters respectively with only a few small outliers. Fig 8 shows these results combined on a single map.

**Fig 9:**
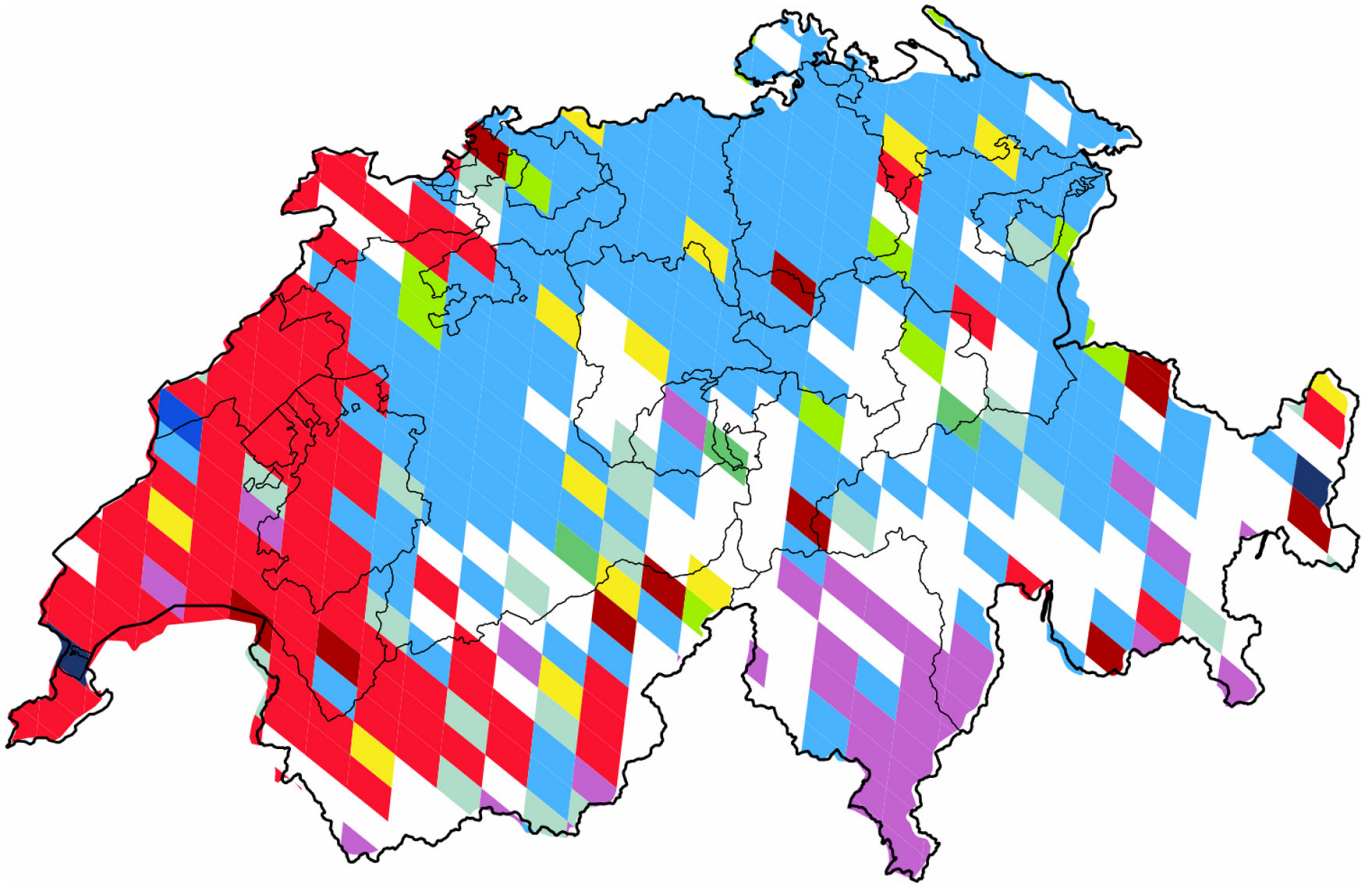
Communities formed in Switzerland. These are clusters formed within the European continent’s regional subgraph, focusing on the single country of *Switzerland*. Each cluster of geo-pixels has a different unique color. The *red* and the *purple* correspond to the communities concentrated in *France* and in *Italy* respectively. The subnational boundaries are marked with thinner lines, depicting the administrative units of this graph.

*Switzerland within Europe* is shown in Fig 9 as a special case. This country presents a unique partition with a cluster covering only partly its own territories, and borders that follow surprisingly closely the regions of its German-, French-, and Italian-speaking communities.

**Fig 10:**
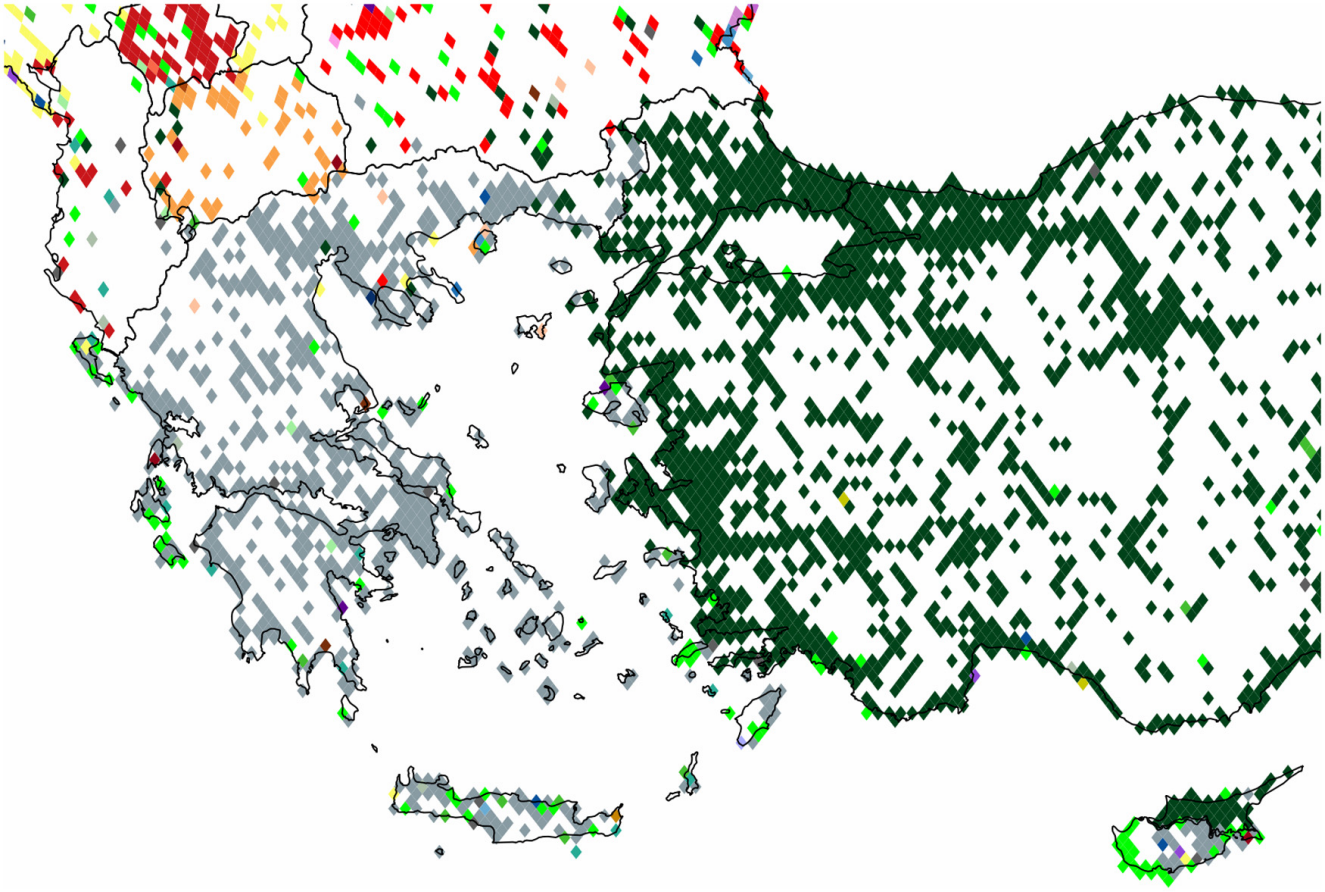
Communities formed in Cyprus and Greece. These are clusters formed within the regional subgraph of the European continent focused on these two countries. Each cluster of geo-pixels has a different unique color. The *gray*, *green* and *dark green* correspond to the cluster with its majority concentrated in *Greece*, the *UK* and *Turkey* respectively. The subnational boundaries of these countries are marked with thinner lines, depicting the administrative units of this graph.

*Cyprus within this region* is effectively divided into northern and southern areas. As opposed to the previous case where only links between the *EU* member states are considered, here, the inclusion of users from *Turkey* makes it possible to see the separation line, connecting the northern area to the Turkish cluster and southern to the Greek one. The western influence on the shores of the Greek islands is still pronounced as it can be seen in Fig 10.

**Fig 11:**
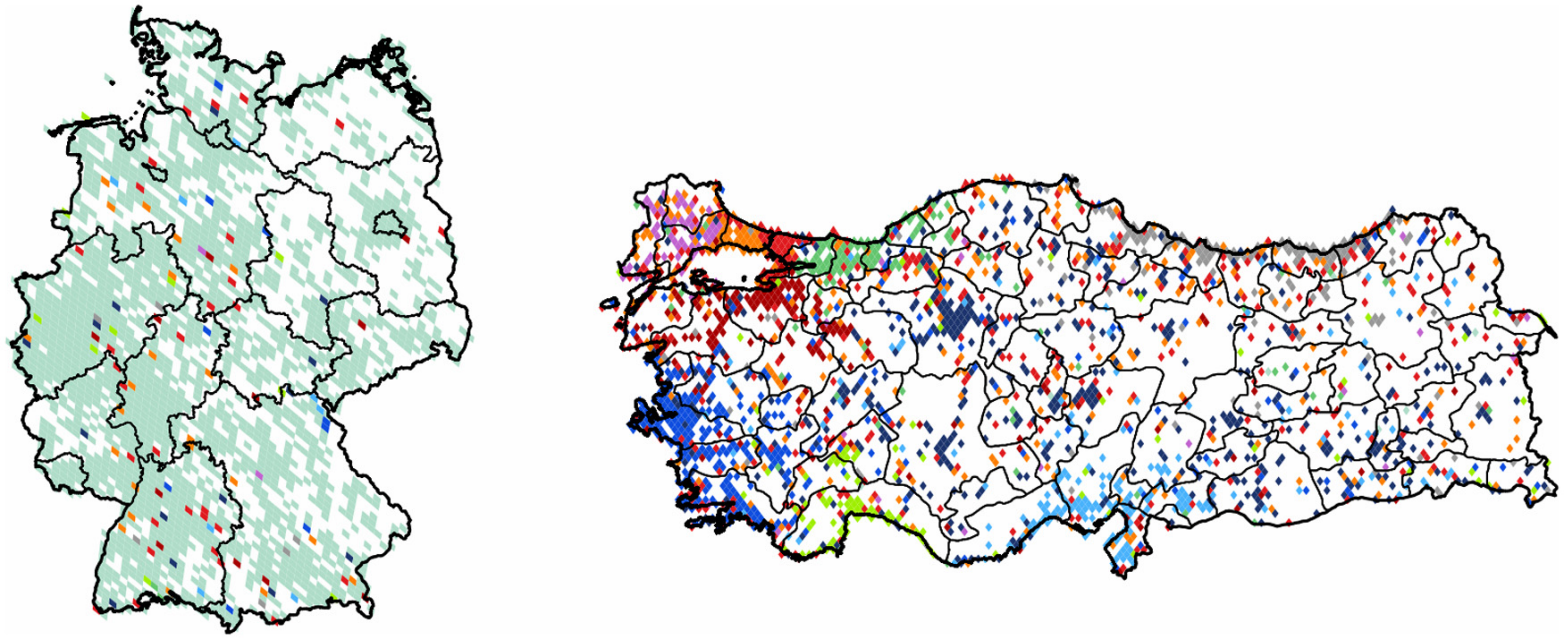
Communities formed in Germany and Turkey. The 4,444 pixels formed 11 clusters with modularty value of 0.3. Each cluster of geo-pixels has a different unique color. The subnational boundaries of these countries are marked with thinner lines, depicting the administrative units of this graph.

The *UK* and *Turkey* are two special cases with clusters that present a strong satellite outreach. The cluster of the *UK* is different in the way that it has its satellites spread throughout all of the countries with weakest presence in the East, *Spain* and *Italy*. This cluster not only has inherent use of the English language but is also a preferred destination of emigrants and students throughout *Europe* and is home of many pop culture figures. On the other hand, the cluster of *Turkey* is heavily present in various parts of *Germany*. We believe that this is a perfect example for the communication patterns as influenced by the ties remaining between immigrants and their original homeland. We studied the subgraph solely formed by the German and Turkish pixels. Fig 11 shows an asymmetric division: while only a few satellite pixels reach *Turkey* from the large and compact cluster of *Germany*, the former is divided into several clusters with outreach to the German area, showing a visible influence in this direction. We note the decrease in the modularity value indicating that the division of German cluster would probably reveal further inherent structure of the graph.

In the following we describe the results obtained for regions within *Europe*. The community structures are shown with the administrative boundaries.

**Fig 12:**
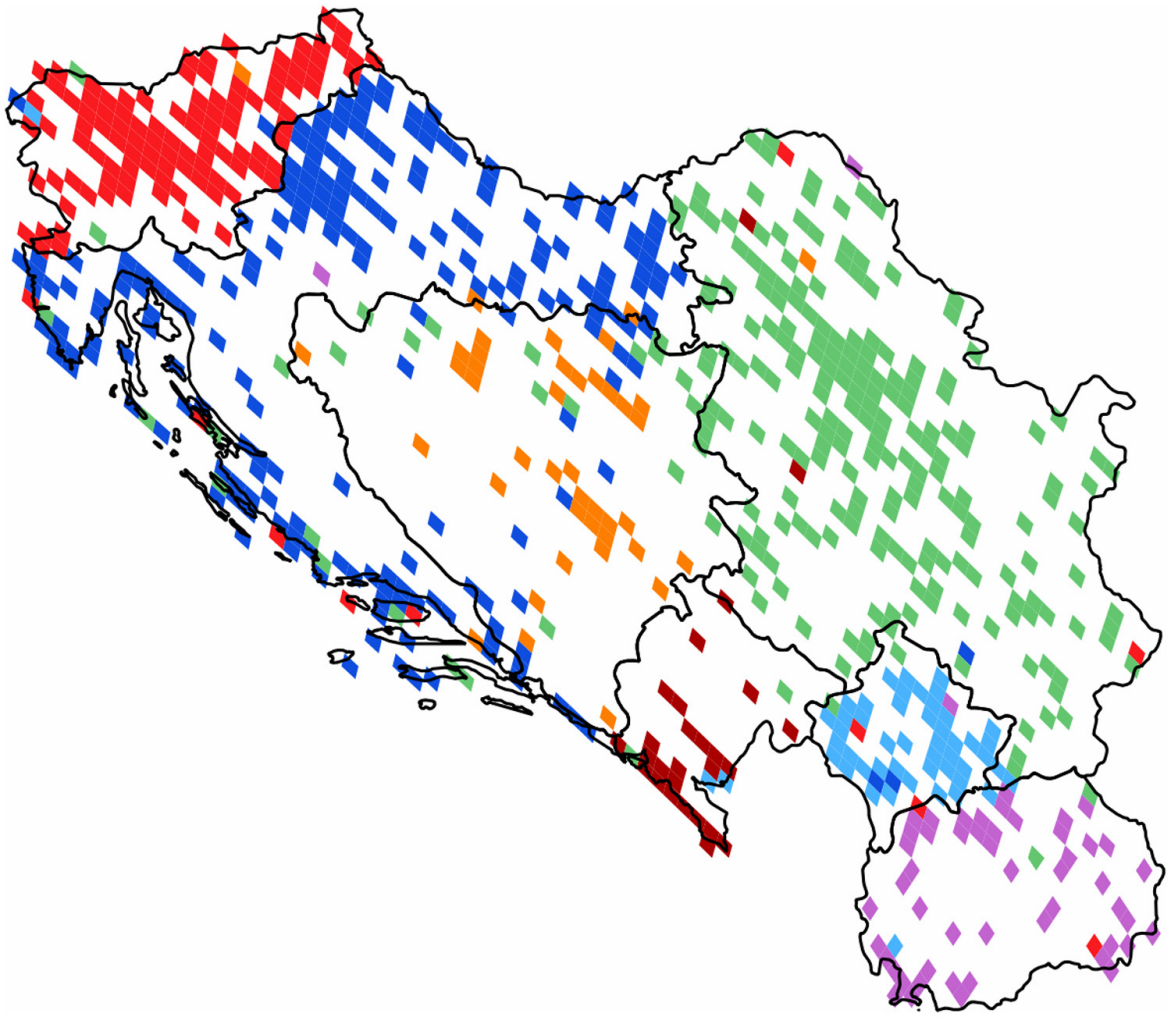
Clustering of the Former Yugoslavia. The 724 pixels form 7 clusters with modularity value of 0.52. Each cluster of geo-pixels has a different unique color. The national borders are marked, depicting the administrative units of this graph.

**The Former Yugoslavia**: This small region has diverse ethnicities living in close local proximity. The historic separating effect of serious local conflicts could possibly be overridden by economical interests. The latter could also be supported by the lack of a strict linguistic barrier in reason of the existence of the South Slavic *Serbo-Croat-Bosnian* language. Although the current administrative borders dived them into seven countries, they formed a single country from 1918 to the end of the century. The found partition has seven cohesive communities and a high modularity value of 0.52. The results are shown on Fig 12. Its clusters are spatially cohesive and follow the post-war administrative borders with rather high accuracy, and with some unbalanced satellite outreach characterizing a few of the clusters. (We note that this area is not well represented in its southern and central parts.)

**The United Kingdom**: Our clustering resulted in a division giving 13 clusters with a modularity value of 0.37, showing a significant cohesiveness within the found clusters. *Northern Ireland* is well separated, and the cluster of *Scotland* covers a relatively large area too. In addition, we can see that this result has approximately the half of the modularity found in [5] for the telephone calls graph. This could either be some kind of a subsampling artifact or it could be indicative of the difference between the intended use of telephones and online media platforms. Latter providing a service for connecting to anyone with similar interests without the necessity of simultaneous bipartite activity, and thus its main limiting factor remaining the language barrier. In case of a single country this barrier is rarely present, and can partly explain the decrease in the spatial cohesiveness of the formed clusters. We note the inclusion of the *Northern Ireland* region, that was missing from their data set. Nevertheless, their results show comparable spatial patterns with a few marked differences. First, they had fourteen clusters and the modularity value of 0.6. Second, a few boundaries are introduced, that are not present in our map. On the other hand, our results confirm the appearance of the newly born region of *Western Crescent*. We find that it has similar extent and shape in our network.

**Fig 13:**
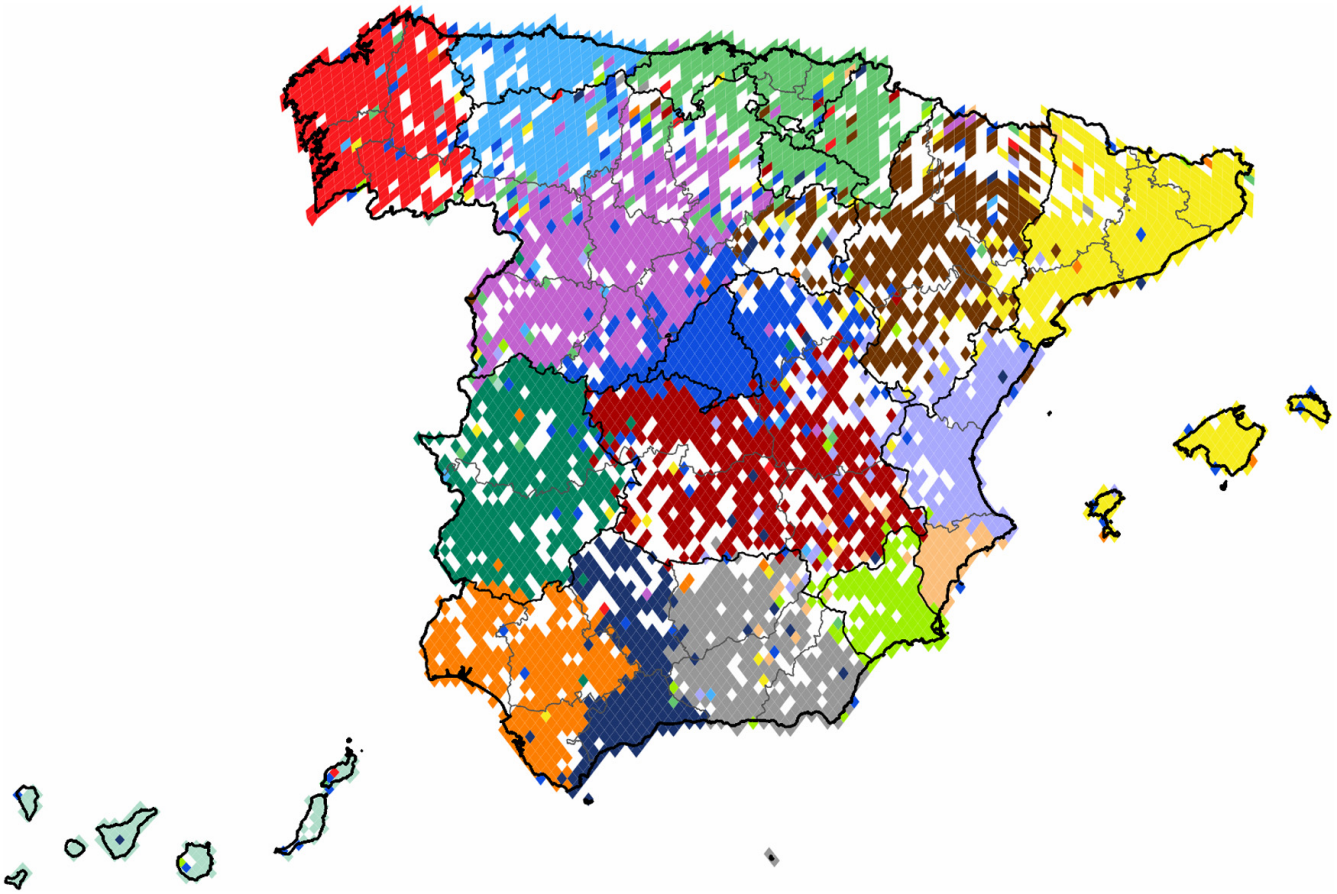
Clustering of Spain. The 4,126 pixels form 16 well defined clusters with modularity value of 0.54. Each cluster of geo-pixels has a different unique color. The subnational boundaries are marked with thinner lines, depicting the administrative units of this graph.

**Spain**: The subgraph of *Spain* is one of the most dense areas with strong user presence in every administrative units. The country is divided into sixteen clusters, a partition characterized by the high modularity value of 0.54. The results are shown on Fig 13. We find cohesive clusters following their respective administrative borders with remarkable precision, but there are also regions that we see divided between more than one clusters. Madrid forms a strong and compact central cluster with an expanded halo and an extensive outreach through evenly scattered satellite pixels. The region of the autonomous community of *Basque Country* forms also a strong and extensive cluster, joined by more than one of its neighbors. Catalonia also forms its own distinct community with the *Balearic Islands*.

**Fig 14:**
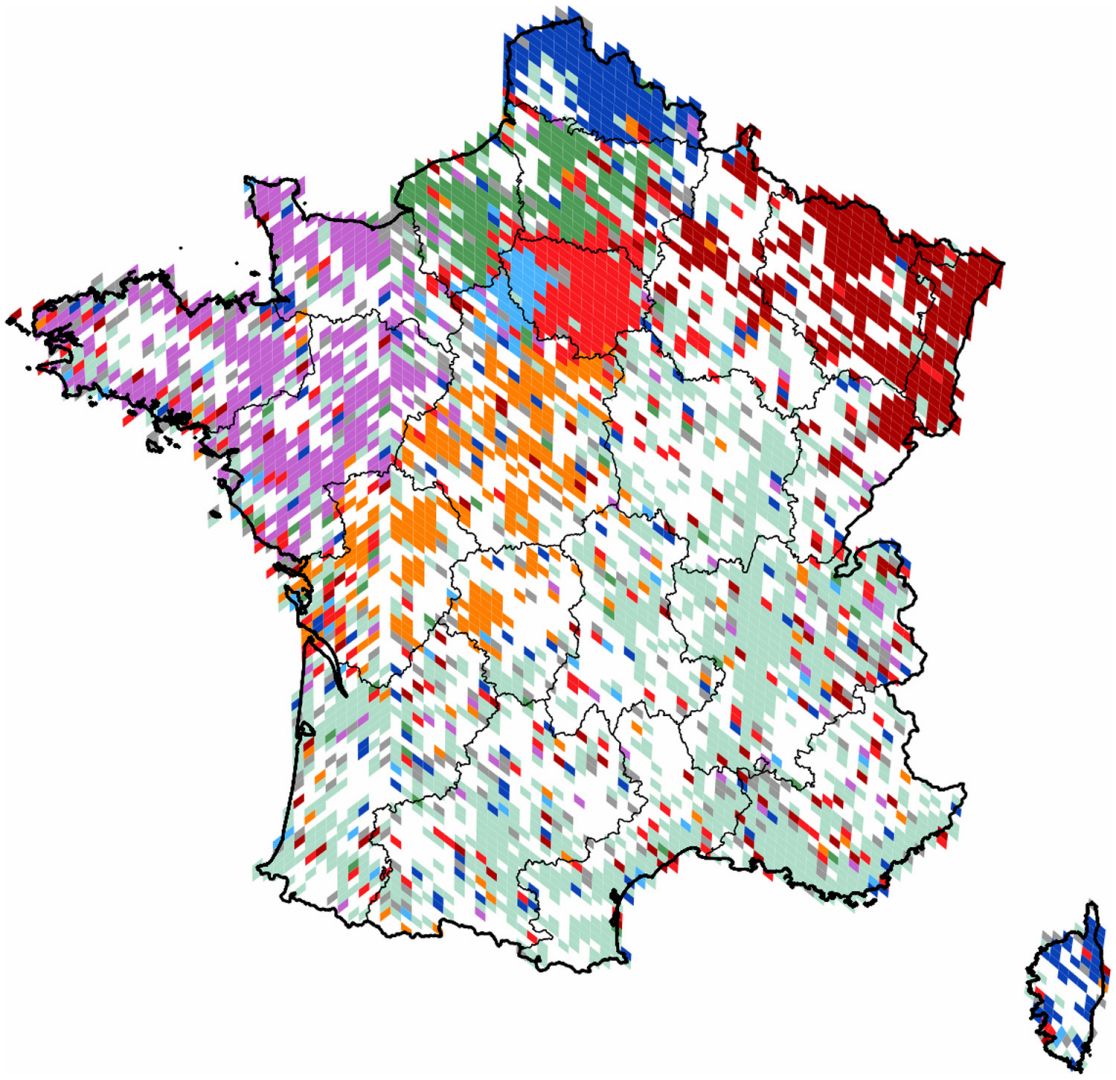
Clustering of France. The 3,488 pixels form 9 well defined clusters with modularity value of 0.3. Each cluster of geo-pixels has a different unique color. The subnational boundaries are marked with thinner lines, depicting the administrative units of this graph.

**France**: We created the regional subgraph connecting the pixels of *Metropolitan France*. As shown on Fig 14, we obtained nine clusters with a modularity value of 0.3. This relatively large country has a density of Twitter users rather small, and concentrated mainly to the metropolitan areas. Accordingly, not all of the clusters show high spatial cohesiveness. The largest cluster of 1,286 pixels is denser on the southern and the eastern regions, but has rare satellites in the rest of the country evenly. The northern part of the region of *Centre* and the western part of the region of *Ile-de-France* form a small but dense cluster with negligible outreach. On the other hand, the rest of *Ile-de-France* forms a community with significant satellite presence in the rest of the country.

**Fig 15:**
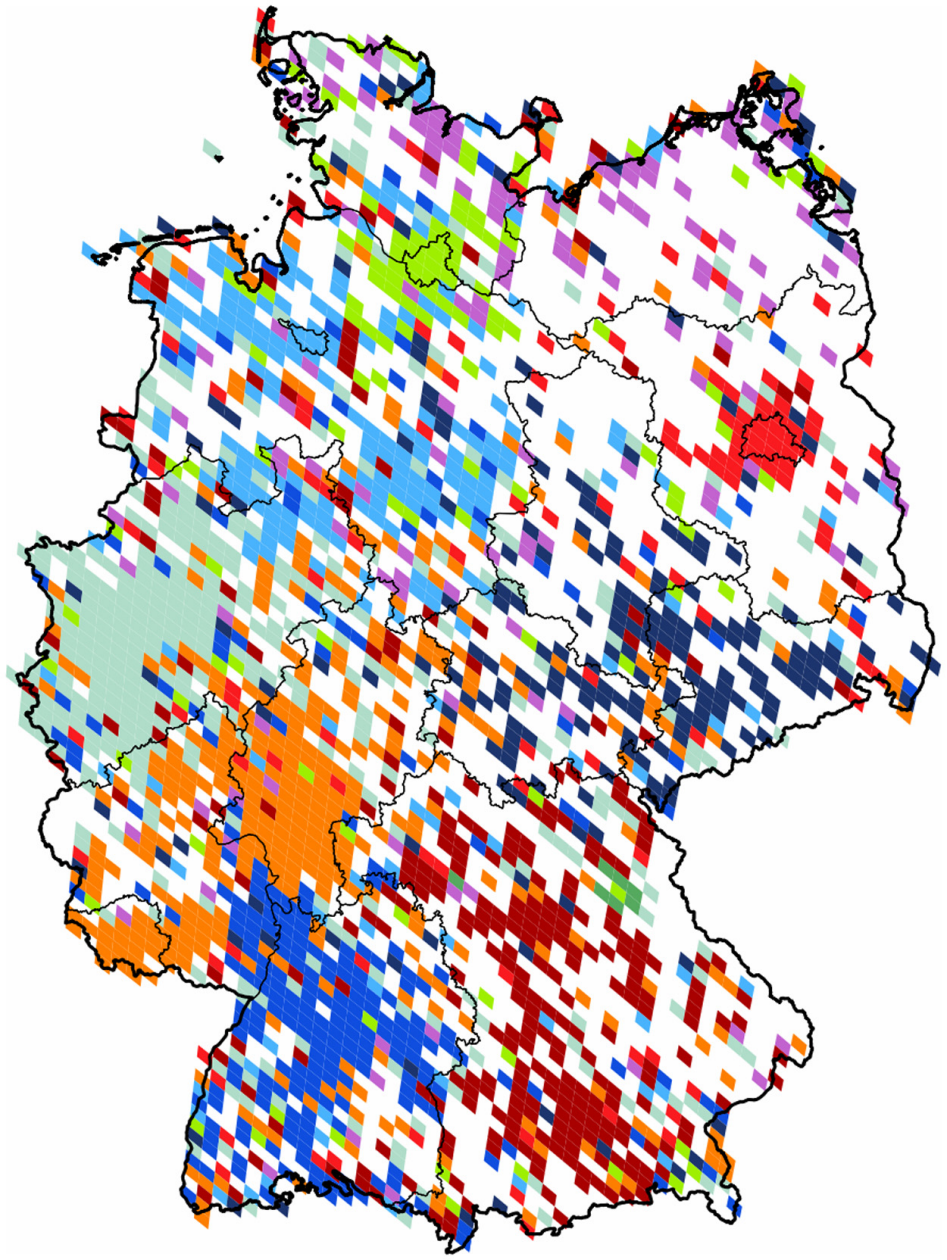
Clustering of Germany. The 2,105 pixels form 10 well defined clusters with modularity value of 0.26. Each cluster of geo-pixels has a different unique color. The subnational boundaries are marked with thinner lines, depicting the administrative units of this graph.

**Germany**: When partitioning the subgraph purely formed by the territory of *Germany* we see a rather scattered projection with sparsely represented areas in the eastern half of the country. This shows that the popularity of Twitter platform is not as high as in other regions of the world. As it is shown in Fig 15 there are nine clusters formed, and an outlier of negligible size, giving the weak modularity value of 0.26. The inhomogeneous use of Twitter is rather concentrated around the main urban areas, and not always covering the majority of the area of their respective states. On the north-eastern part we have a small, but dense cluster around the city of *Berlin*, and on the northern areas, a similar cluster around *Hamburg*.

#### Community Structures in Asia

In general the coverage of this area is not homogeneous, mainly due to the very diverse levels of popularity of Twitter in the different countries. Although similar micro-blogging services are ahead of Twitter in popularity in the *People’s Republic of China*, since the access to Twitter has been effectively blocked in the country, in accordance with previous findings [18], our measurements still show activity. Despite the severe censorship present we see evenly spread users, but the representation is weak and geographically scattered. *India* shows similar underrepresentation, but the service is not locally blocked. In contrast, one of the most active Twitter zones worldwide is located in this region as the *Philippines* and *Malaysia* are both highly active and well represented countries. Here we show the result of three subgraphs. First we study the *Southeast Asia* with *China* and *Japan* included. Then we show the clustering results for the single-country subgraphs of *India* and of *Japan*.

**Fig 16:**
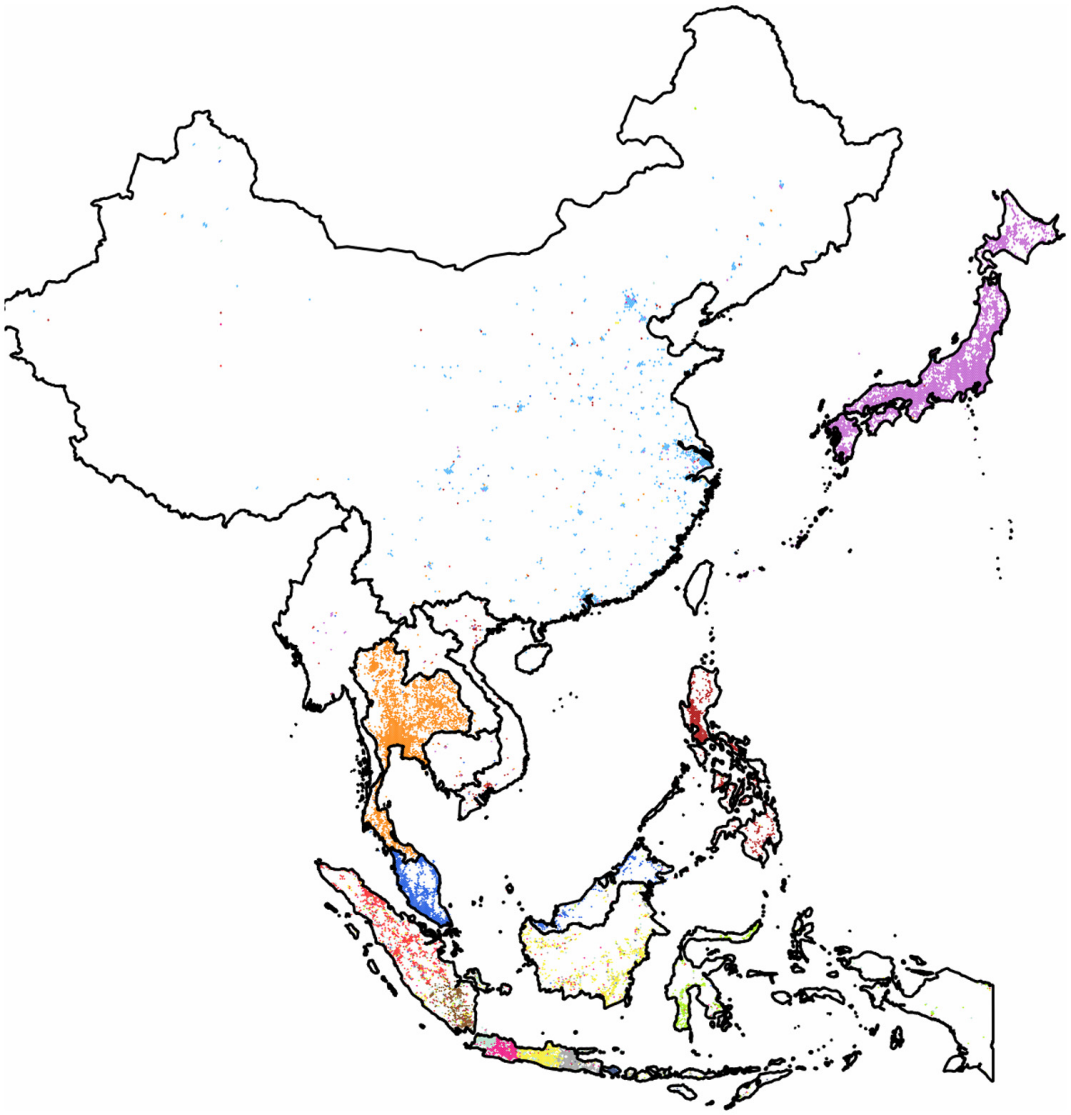
Clustering of the region of Southeast Asia with inclusion of China and Japan. The 13,033 pixels form 13 clusters with a high modularity value of 0.7. Each cluster of geo-pixels has a different unique color. The national borders are marked with thinner lines, depicting the administrative units of this graph.

**Southeast Asia with China and Japan**: This region has the fourth largest subgraph in our set. Its partition of thirteen clusters has a modularity value of 0.7. The results can be seen on Fig 16. Communities are well localized within the administrative borders of countries, or showing their more detailed internal structure in a few cases. We rarely see clusters containing pixels from more than a single country. A single cluster covers effectively the whole of the Chinese pixels and it also includes the majority of the pixels of the small state of *Brunei* situated on the neighboring island of *Borneo*.

While this is as far as this cluster’s outreach goes, we note that the Chinese territory contains at least one satellite pixel from each of the other clusters with a few of them having a more significant presence. *Japan* is densely occupied. With insignificant presence of satellites from other communities it forms its own cohesive cluster that is the largest one with more than 3,000 pixels and with a relatively week outreach. While *Malaysia* forms a cluster with a few satellites evenly spread, the Philippines is covered by a cluster that has a more pronounced outreach.

**Fig 17:**
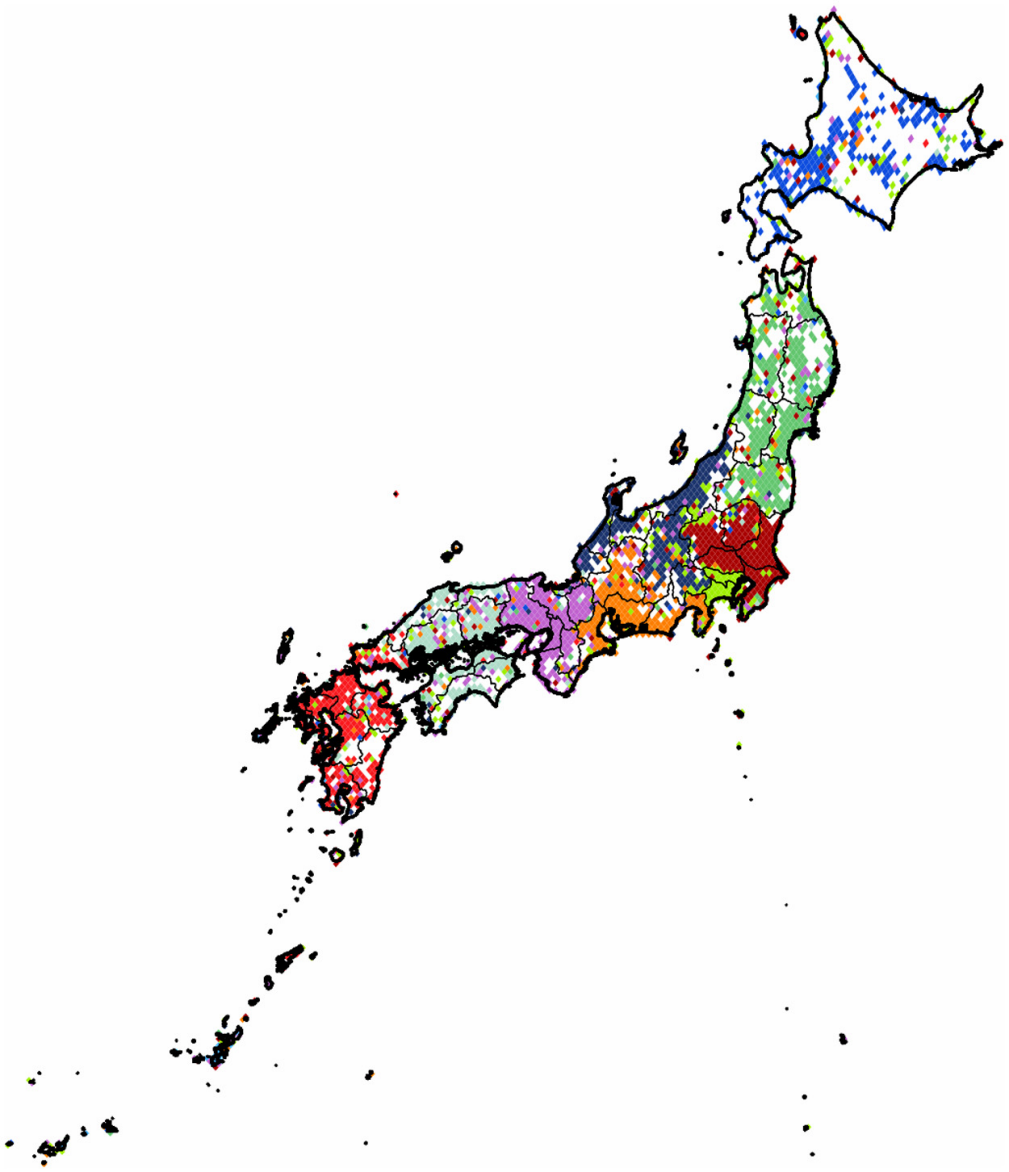
Clustering of Japan. The 2,955 pixels form 10 clusters with a modularity value of 0.19. Each cluster of geo-pixels has a different unique color. The subnational boundaries are marked with thinner lines, depicting the administrative units of this graph.

**Japan**: When we partition the subgraph of *Japan* it almost constitutes the second-level clustering of one of the communities found in the above discussed large region. This graph falls into the small category, and we obtained a partition with modularity value of 0.19. The results can be seen on Fig 17. The country is cut into ten parts and their boundaries loosely follow the administrative subunits. The region surrounding *Tokyo* has its own smaller cluster with attached part from the west northern extreme of *Kanto* and visible satellite presence throughout the country.

**Fig 18:**
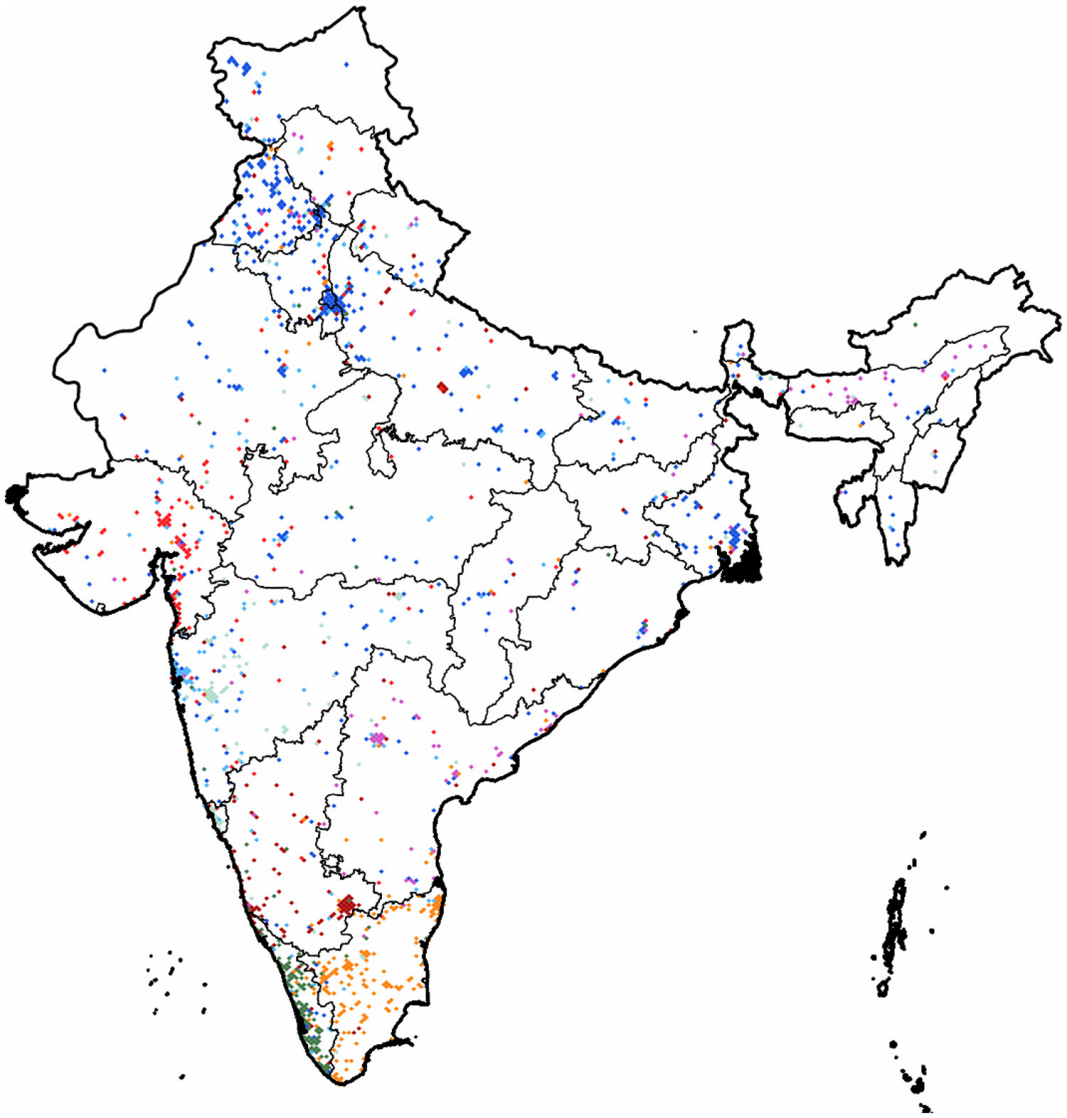
Clustering of India. The 1,485 pixels form 8 clusters with a modularity value of 0.36. Each cluster of geo-pixels has a different unique color. The subnational boundaries are marked with thinner lines, depicting the administrative units of this graph.

**India**: This is the second smallest regional graph that we partitioned. It has a spatial structure similar to the one seen in the case of the Chinese coverage with activity rather present around a few concentrated urban areas. The partition reveals eight communities with modularity value of 0.37. The results are shown on Fig 18. The largest cluster has its denser core at *New Delhi* and *Chandlgarh* and it occupies most of the active pixels of the state of *Punjab* while reaching all the way to *West Bengal* and having an outreach in most of the country as well, leaving out the southeastern state of *Tamil Nadu*. The smaller clusters can be divided into two size categories. The medium ones are around 150 to 200 pixels large, while the two smallest are around only 110 pixels.

## Conclusion and Outlook

While political maps are slowly changing throughout history our analysis revealed how recent self-formed communities of active “netizens” partition the geographic space. As opposed to previous works of similar methods, here we studied a spatially embedded online communication network. As the social ties of the Twitter platform are not constrained by the limitations of local telecommunication providers its geographic coverage reaches a considerably higher level. Relying solely on topological information enabled us to draw the geographic partitionings without using any meta-information on the social or linguistic properties of the individual user accounts. The communities form more or less cohesive units similarly throughout four different continents. The *goodness* of the clustering as measured by the modularity was better in each regional network than the goodness of the administrative clustering. The fact that their difference was not extreme shows that they overlap substantially. A randomized rewiring of the *UK* graph showed how the spatial proximity is not enough to get such projected structure. We showed examples of linguistic and historic separations like e.g. the communities formed in *Switzerland* or *Canada*. We also found spatial fingerprints of separation in local conflict zones like e.g. in *Cyprus*, *Spain* or the region of the *Former Yugoslavia*. Uniting forces of globalization as reflected in the far-reaching influential clusters of *Turkey* and of the *UK* within *Europe*, or the touristic zones along the coastlines of the popular destination of the *Greek islands* that are heavily colonized by satellites from foreign communities. Segmentation of regions of high density of population into urbanized areas can be seen at various places in the *Americas*. We showed partitioning of a large *Asian* subgraph as well, where the spatially scattered but nonetheless non-negligible *Chinese* community exists despite the local access ban of Twitter. Despite this rich coverage, our research had to use limited resources with regards to both the measurement capacity and the data processing computer power. Nonetheless, these results show that the publicly available resources for the academic research community can be sufficient to uncover well detailed spatial patterns of human interaction.

We propose an outlook as we next explore some directions in order to solve some of the shortcomings in future works. Further exploration of this spatial social graph by using better algorithms and or better data sets could give us new insights. The same method could be applied to a wider set of mid-range or country-level regions, or we could advance by taking a step away from the methods initiated by studies of restricted social graphs. Refocusing to the topology of the global graph and discarding the preset limitation of the different cultural or geographic boundaries, a straightforward hierarchy of clusters could be determined through a few levels of spatial segmentation. Starting from a global graph of geo-pixels further partitioning of resulting clusters could reveal the finer details. This would require either more computational capacity or a more efficient optimizing algorithm. Without any of these only coarser pixel choice could be used. Another obvious future direction of research could be the study of a new version of the Twitter graph. However, the entire graph is still not freely available to the general public, and the regulations of the public API are frequently changing over time. Hence the collection of new geo-users and their spatially embedded connections seams to be out of reach. Instead, we have started to explore two directions. First, the used weighted links currently inferred only from follower or friendship links could be adjusted based on communication measurements, like directed messages or replies sent between the geo-users. Second, the used set of nodes could be complemented by including the users without prior geographic information. Previous studies reported promising results in this direction [19–22]. This study would be probabilistic in nature, as the analysis would use statistically inferred locations based on the connections between users of known and unknown location.

## Supporting Information

S1 File This file contains complementary information about materials, methods and results. Figure A in S1 File: flowchart of greedy modularity optimization algorithm. Figure B in S1 File: flowchart of randomized greedy modularity optimization algorithm. Figures C-E in S1 File: histograms of number of users per pixel in each region considered in the main text.
(PDF)

S2 File This file contains the regional data sets. After decompression, one file per region: <region>.dat in S2 File contains in a multicolumn text format the data sets necessary for reproduction of the presented community projection results. Additional two-column files: <region>-hist.dat in S2 File contain data sets corresponding to the histograms of number of users per pixel in each region considered in the main text. (These histograms are depicted on Figures C-E of S1 File. S2 File data structure is described in the last section of S1 File.)
(ZIP)
