# Supplementary material for: Spatial Fingerprints of Community Structure in Human Interaction Network for an Extensive Set of Large-Scale Regions
Source: PLoS One. 2015 May 20;10(5):e0126713. doi: 10.1371/journal.pone.0126713 (PMC4439170; doi:10.1371/journal.pone.0126713)
Supplement: S1 File — Figure A in S1 File: flowchart of greedy modularity optimization algorithm. Figure B in S1 File: flowchart of randomized greedy modularity optimization algorithm. Figures C-E in S1 File: histograms of number of users per pixel in each region considered in the main text. (PDF) [file pone.0126713.s001.pdf]

SUPPLEMENTARY INFORMATION

Spatial Fingerprints of Community Structure in Human Interaction Network for an Extensive Set of Large-Scale Regions

Zsfia Kallus[1*], Norbert Barankai[1], Jnos Szle[1], Gbor Vattay[1]
1 Department of Physics of Complex Systems, Faculty of Science, Etvs Lornd University, Budapest, Hungary
∗ E-mail: kallus@complex.elte.hu

1 Modularity and modularity clusterization

Modularity as proposed by Newman and Girvan [1] is a popular measure that tries to quantify the goodness of a given partition of vertices of a graph that presumably gives the community structure of a network represented by that graph. The measure assigns a simple number to every given partition of vertices. The idea is that the partition of vertices of graph $\mathcal{G}$ with maximal modularity (compared to other partitions of the same graph) represents the 'true' community structure of the network under consideration. A partition of vertices is a mutually disjoint series $V_1, \ldots, V_l$ of groups of vertices such that every vertex belongs to one and only one group. These groups are called clusters. Let e_{ij} be the fraction of edges that connects vertices of V_i and V_j. The total fraction of edges that connect to a vertex in the cluster V_i is $\sum_j e_{ij}$. Intuitively, e_{ij} is the probability in $\mathcal{G}$ that a randomly chosen edge connects vertices one from V_i and the other from V_j. Similarly, a_i is the probability in $\mathcal{G}$ that a randomly chosen edge connects to a vertex of V_i. If the graph has no structure, that is the edges are placed randomly between the nodes of the graph, e_{ij} is about $a_i a_j$. This motivates the following definition of the modularity Q of the partition consisting of the vertex groups $V_1, V_2, \ldots, V_n$:

$$Q(V_1, V_2, \ldots, V_n) = \sum_{i=1}^{n} (e_{ii} - a_i^2).$$

For random graphs Q is close to 0. Its maximum value is 1. The strategy to detect communities based on Q seems to be simple - one only has to find the partition such that its modularity is maximal among all partitions of the same graph. Unfortunately, this task is practically impossible for large graphs: the necessary time to find the exact solution of the problem is astronomical (see [2] for details). To cope with the situation, numerous heuristic methods have been proposed. All of them try to approximate the exact solution. The so called greedy modularity clustering has been proposed by Newman [3]. For a graph with N vertices, the algorithm surely stops after N steps. The input of the algorithm is the graph itself and an initial partition. Usually the initial partition is the finest of all possible: it contains N groups, each group has only one member, that is each vertex forms it own cluster. In each algorithmic step, we select two vertex clusters that give the highest increase in modularity if they are merged into one single cluster. The change in modularity when V_i and V_j are merged is

$$\Delta Q = e_{ij} - 2a_i a_j.$$

The algorithm stops when any possible merges would result in a partition with decreased modularity. See Fig. A for a simplified flowchart of the algorithm. This algorithm is very fast but for large graphs gives only a poor approximation. The reason for that is the rigidity of the choice made in each algorithmic step: a journey in the space of possible partitions that have an ever increasing modularity usually results only in a local maximum, not the needed global one. To handle this, some randomization has to be incorporated into the process. We follow the work of Ovelgönne et al. [4] and use their randomized greedy modularity algorithm. The rough details of the algorithm are as follows. The input is again the graph $\mathcal{G}$ and an initial partition of it. Furthermore, we have a positive integer m. At each algorithmic step we

choose randomly m clusters (or less, if the number of clusters is less then m) and collect them and their neighboring clusters to a set $\mathcal{K}$. After the calculation of the fractions e_{ij} and a_i among the members of $\mathcal{K}$ we select two clusters that give the highest increase in modularity if they are merged into one single cluster. We merge these clusters, save their indices and the change in modularity (ΔQ_k in the kth step). We perform the steps until there is only one cluster that contains all vertices of the graph. Using the modularity Q_0 of the initial partition of the vertices and the series $\Delta Q_1, \dots, \Delta Q_l$, the modularity of the partition obtained at the end of the kth step can be calculated by

$$Q_k = Q_0 + \Delta Q_1 + \dots + \Delta Q_k.$$

After the maximum of the series $Q_0, Q_1, \dots, Q_l$ has been found, the corresponding partition of vertices can be reconstructed from the initial partition and the saved indices of the clusters merged in each step. Thus the final result of one running of the algorithm is a (usually) new partition with an increased modularity compared to the initial one. See Fig. B for a simplified flowchart of the algorithm. To get a good approximation the algorithm has to be run numerous times with the same input. Finally, the partition with the highest modularity gives the approximation of the original optimization problem. For further details and technicalities see [4] and [5].

2 Adjusted Rand index and normalized mutual information

Given a finite set of elements of a set, their partition is a mutually disjoint series of subsets such that every element is in exactly one of these subsets. The need of comparing two partitions and expressing their difference in a single number led to the introduction of numerous difference measures [6]. Our paper used the *adjusted Rand index* and the *normalized mutual information* to quantify the difference between the clustering of geographical pixels by administrative regions and by our method using the topology of their Twitter connections.

The Rand index [7] is defined as follows. Let $\{X_1, \dots, X_n\}$ and $\{Y_1, \dots, Y_m\}$ be two partitions of the same set $\mathfrak{A}$ that contains N elements. We denote the partitions by X and Y. Let $P(X, Y)$ be the number of pairs of distinct elements of $\mathfrak{A}$ that belong to the same class in both partitions. Let $Q(X, Y)$ be the number of pairs of distinct elements of $\mathfrak{A}$ that do not belong to the same class in each partition. The Rand index is defined as

$$\mathcal{R}(X, Y) = \frac{P(X, Y) + Q(X, Y)}{E},$$

where E is the number of distinct pairs formed by the elements of $\mathfrak{A}$. Unfortunately the Rand index does not vanish when averaged over pairs of random partitions. To cure this anomaly, the adjusted Rand index had been introduced in [8]. Define E_{ij} as the number of distinct pairs formed by elements of $X_i \cap Y_j$. Let K_X and K_Y be the number of those distinct pairs whose members are in the same cluster of the partition $\{X_1, \dots, X_n\}$ and $\{Y_1, \dots, Y_m\}$, respectively. The adjusted Rand index can be calculated by

$$\mathcal{R}_{\mathrm{ad}}(X, Y) = \frac{\sum_{i=1}^{n} \sum_{j=1}^{m} E_{ij} - K_X K_Y / E}{(K_X + K_Y)/2 - K_X K_Y},$$

and it gives the normalized difference between the Rand index of two partitions and their expected value. The assumed distribution is the generalized hypergeometric distribution.

The normalized mutual information [9] is defined as follows. Consider again the two partitions $\{X_1, \dots, X_n\}$ and $\{Y_1, \dots, Y_m\}$ of the set $\mathfrak{A}$. Both partitions define a probability measure. Define $P_X(i)$ as the probability of finding a randomly chosen member of $\mathfrak{A}$ in X_i. The same definition applies to probabilities $P_Y(j)$ if one replaces X_i with Y_j. The information content of probability distributions P_X and P_Y are described by the information entropy H_X and H_Y:

$$H_X = -\sum_{i=1}^{n} P_X(i) \log_2(P_X(i)) \qquad H_Y = -\sum_{j=1}^{m} P_Y(j) \log_2(P_Y(j)).$$

The smallest value of the information entropy is zero and, in the case of partitions, it corresponds to the trivial partition of $\mathfrak{A}$, that contains only one cluster. The largest value of the information entropy among various partitions of the same set is $\log_2(N)$ and it corresponds to the finest partition when all the members of $\mathfrak{A}$ are in different clusters. To compare the two partitions in an information theoretic framework, we need another distribution $P_{XY}(i,j) = |X_i \cap Y_j|/N$ that gives the probability that a randomly chosen node is in $X_i \cap Y_j$. Then, the mutual information I_{XY} of the two partitions is defined as

$$I_{XY} = \sum_{i=1}^{n} \sum_{j=1}^{m} P_{XY}(i,j) \log_2 \left(\frac{P_{XY}(i,j)}{P_X(i) P_Y(j)} \right).$$

This measure tries to quantify the volume of the shared information of the distributions. For independent partitions, the mutual information turns to be equal to zero. If the two partitions are the same, the mutual information is equal to the information entropy of that partition. This suggests a redefinition by normalization resulting in the normalized mutual information:

$$\mathcal{S}_{\mathrm{n}}(X,Y) = \frac{I_{XY}}{\sqrt{H_X H_Y}}.$$

3 Number of users in geographical pixel regions

As more than half of the Earth's population lives in highly urbanized areas the spatial distribution or density of people is inherently highly inhomogeneous. Working with the subset of people using public Twitter account can further distort this distribution. As central parts of cities has a much higher density the equal area pixels will have less of high user numbers and more of low user numbers. Hence the histograms depicting the distribution of user numbers per pixel in each of the regional graphs in Fig. C-E show the expected behavior. We note that this does not result in a distortion of the clustering results.

4 Complementary details of the reprojected regional clusters

Here we give details complementing the descriptions presented in Sec. *The projected regional clusters* in *Results and Dicussion.*

North America

We note that when partitioning a subgraph of the combined area of the *US* and of *Canada*, the latter forms its own cluster. As in the similar case of the subgraph of the combined *US* and *Mexico*, there are only a few satellite pixels swapped between the countries, mainly from the neighbor clusters on the two sides of the respective borders.

Canada

Newfoundland and *Labrador* forms a cluster with *Nova Scotia* and *New Brunswick* on the eastern extreme with only a few satellite pixels spread from another cluster. *Quebec* forms its own cluster but also swaps a few satellite pixels with its western neighbor. Its center of gravity is divided between the cities of *Montreal* and *Quebec. Ontario* is the exception with having four different clusters. One is formed on the shore to the south of the city of *Ottawa.* A smaller cluster is formed around *Toronto* that is also intertwined on its northern half by its neighbor. This cluster is formed around the *Toronto* area, following the border with the *US.* The remaining western territories of *Ontario* are clustered together with the pixels of *Manitoba.* On the other hand, *Saskatchewan* is joined together with *Alberta.* This larger cluster

is rather sparse, but shows denser areas around the cities of *Calgary* and *Edmonton.* Few satellite pixels reach the western cluster of *British Columbia.* The pixels of this cluster are mainly concentrated around the cities of *Vancouver* and *Victoria.* These two clusters have a few satellite pixels in the rarely occupied *Northwest Territories* and *Yukon.*

The Continental United States

Main, *New Hampshire*, *Vermont*, *Massachusetts*, *Rhode Island* and *Connecticut* form a cluster along with the eastern half of the state of *New York* and north-eastern half of the state of *New Jersey.* The western part of *New York* forms a cluster in itself, and the south-western part of *New Jersey* is part of a cluster with *Delaware* and the state of *Pennsylvania.* *Washington, D.C.*, *Maryland* and *Virginia* form a cluster with a somewhat fuzzy border at its south-western corner, while *West Virginia* is joined to *Ohio.* *North* and *South Carolina* form a well defined cluster, just like *Alabama* and *Georgia.* *Florida* forms a cluster on its own just like *Michigan*, while *Mississippi* is joined to *Tennessee* and *Kentucky*, with a border of their cluster loosely following the administrative regions. These were the regions with the most dense populations.

The central part of the map shows more empty pixels. On the northern part there is a large cluster formed by *Wisconsin*, *Minnesota* with *North* and *South Dakota.* *Iowa*, *Illinois* and *Indiana* are joined together, loosing a small area from the central part of *Iowa* and from *Illinois* at the border shared by *Missouri.* This big, central cluster of *Missouri* also includes *Nebraska*, *Kansas*, *Oklahoma* and *Arkansas.* At the South with clear borders corresponding to administrative regions *Texas* and *Louisiana* are joined together. This marks the end of the well partitioned eastern part of the *US.*

For further detail of the partitioning we performed a second-level clustering on the large western cluster. There is a strict cluster formed by *New Mexico* and *Arizona* and one by *Oregon.* The northern half of *Idaho* is joined to the cluster of *Montana* and the state of *Washington*, while its southern half is joined in a cluster with *Utah.* *Wyoming* is rather sparsely represented and the majority of its pixels are in the cluster of *Colorado.* *Nevada* forms its own cluster, with slight penetration to its northern neighbor. As seen *Alaska* is rather sparsely populated, forming its own cluster, with a few satellite pixels. *Hawaii* has also a small number of occupied pixels and has mostly satellite pixels with most of them connected to *Alaska* and *California.* The state of *California* is a special case. The northern part is divided between the clusters of *San Francisco* and of *Sacramento.* In the center, the cluster of *Fresno* reaches down to *Bakersfield.* The compact but dense cluster of *Los Angeles* is neighboring the similarly sized cluster of the cities of *Riverside* and *San Bernardino*, reaching all the way to *Mexicali* on the shore. Enclosed by them, on the shore we find the smaller but well defined cluster around *San Diego* with *Tijuana* and *Oceanside* at its extremes.

South America

In the west, *Chile* forms its own dense cluster. Although their densities get progressively smaller, a separate community is formed by the majority of the pixels of *Ecuador*, *Peru* and *Bolivia.* On the south, *Argentina* and *Uruguay* form a single cluster, with significant outreach to the north-western clusters. *Paraguay* forms a rather compact cluster with few satellites evenly distributed. *Colombia* and *Venezuela* form two separate clusters, the former being more cohesive, while the latter having the highest influence in the region with its satellites present in all other countries, except its eastern neighbors. Those three smaller northern countries, on the other hand, *Guyana* and *French Guyana* and *Suriname* are each part of small Brazilian communities. The first is joined to the cluster of its southern neighbor pixels, the second included in the cluster formed in its immediate eastern neighborhood, concentrated around the island of *Marajo*, at the mouth of the *Amazon* river. The third is joined to the relatively larger central cluster formed mostly around the big cities of *Brasilia* and *Goiania* and with even satellite presence in *Brazil.* The largest clusters are formed on the eastern shore. Two cohesive clusters formed on the north and the

east, and the most dominant cluster in the center of this area. This latter not only contains both of the active cities of *Sao Paolo* and *Rio de Janeiro*, it also reaches the sparsely represented south-western states and all the way to the state of *Alagoas* as its northern extreme.

Europe

The European Union

Northern Europe along with *Estonia* and *Bulgaria* form a cluster with only a few remaining satellites. *Latvia* and *Lithuania* are joined to *Portugal*, giving one of the most geographically disjoint communities. *Spain* forms a compact and very dense cluster, with most of its scattered satellites located in *Germany* and *France*. *Netherlands* and *Belgium* form a compact community with satellites heavily present in *France*, *Germany* and the western part of *Austria*. *Ireland* forms its own small cluster with negligible number of scattered pixels. *Italy* forms its own community, with weak presence in *Portugal*, *Germany* and *Central Europe*. *France* also forms a cluster including *Luxembourg* and evenly scattered additional satellites. *Germany* is divided with a fuzzy limit between the northern and eastern territories forming their own communities, while the *Check Republic* and *Slovakia* form a cohesive unit. Single countries forming a compact cluster are *Poland*, *Slovenia*, *Croatia*, *Hungary* and *Austria*, the latter with weak presence in *Germany*.

The European Continent as a whole

Disproportionately large clusters are formed by Germany and by Russia. The first reaching all the way through the central region - covering *Poland*, *Austria*, *Hungary*, *Slovenia*, *Croatia*, *Bosnia and Herzegovina*, *Montenegro*, *Serbia*, *Macedonia* and *Bulgaria* - and the latter containing mostly the eastern countries - covering *Ukraine*, *Belarus*, most of the *Baltic States*, *Slovakia*, the *Czech Republic*, *Cyprus* and a few satellite pixels. On these large clusters we performed secondary clustering giving eleven and eighteen new clusters respectively with only a few small outliers.

From the first level of the partitioning we get the clusters of the following single countries: *Spain* with satellites in *France*, *Germany* and *Switzerland*; *Italy* with similar outreach; *Portugal* forming a compact unit, and *Greece*. Clusters formed by multiple countries are the northern cluster of *Scandinavia* with pixels of *Iceland*; *Netherlands* joined to pixels of *Liechtenstein*, *Luxembourg* and a few satellites in *South Belgium* with heavy presence in *France*, *Germany* and the central region; and *Greece*, weakly present in other countries but presenting the division between shores with foreign satellites and central areas of its own cluster. The cluster of *France* occupies the remaining majority of the pixels of *Belgium* and *Luxembourg* with satellites heavily present in *Portugal* and weakly scattered in other countries. The cluster of *Greece* has a few satellites in *Germany* and includes the pixels of *South Cyprus*.

In addition, we studied the subgraph solely formed by the German and Turkish pixels. As a result, we get a partition with a modularity value of 0.29. While only a few satellite pixels reach *Turkey* from the large and compact cluster of *Germany*, *Turkey* is divided into ten rather scattered smaller clusters, mostly following large urbanized areas. What is interesting, is that most of these clusters have an outreach into the German area, having a combined effect far larger in this direction than the reverse influence coming from the German cluster. We also note that the satellite pixels do not form a cohesive region within *Germany*, they are rather spread evenly throughout the country. We note that Turkish local censorship [10] following the temporary complete access ban on Twitter in 2014 [11] has been implemented only after the period of our data collection, having no effect on our results.

Former Yugoslavia

The community of *Slovenia* is densely represented, compact, with a few satellites on the shores of the *Croatia*, and one or two peripheral pixels joined from *Serbia*, *Kosovo* and *Macedonia*. The cluster mainly

concentrated in *Croatia* has outreach in *Bosnia and Herzegovina* and only one or two pixels reaching *Serbia* and *Kosovo*. The cluster of *Bosnia and Herzegovina* is sparse but compact, and only has a single pixel in each of the areas of *Slovenia*, *Serbia* and *Croatia*. *Kosovo* clearly forms its own cohesive community, having only one satellite pixel in *Macedonia* and two in *Montenegro*. The area of *Montenegro* forms its own cluster, is also sparsely represented and has active pixels mostly in the south. On the other hand, *Serbia* has great coverage, and its cluster has the most outreach with satellites present on the shoes of *Croatia*, in the northern part of *Bosnia and Herzegovina*, and only one or two satellites in the rest of the countries. The cluster of *Macedonia* is sparse, compact, following the administrative border like the other communities. It only has single satellites in *Kosovo*, *Serbia* and *Croatia*.

The United Kingdom

The total number of clusters found is thirteen. The administrative regions of *England*, *Scotland*, *Wales*, *Northern Ireland* are more or less well separated by well defined boundaries, the only exceptions being *Wales* and *England*. These two share a somewhat fuzzy separation as compared to the administrative border. The only country having been divided into smaller units is *England*. The region of *Greater London* has a strong influence shown by a cluster that has satellite pixels being spread in all of the other units. The rest of the resulting communities have only a negligible amount of spread into other territories. Moreover, this is the cluster having the most distorting effect on its neighboring regions: it divides the *South East* into three parts, occupying the middle with an expansion of its boundaries to the north and the south as well. The remaining parts of this region form two separate clusters. The south-eastern cluster is expanded slightly to the *East Midlands* region, while the cluster to the west of the *Greater London* area is slightly elongated to the south-eastern part of the south-eastern region of *England*. Next, the rest of the *South West* region of *England* has a well defined boundary and a geographically compact area. The region of *East Midlands* in *England* is divided into three parts. While its extreme part of the southern half is attached to the south-eastern cluster, its remaining territories form a unit cluster. Its central part, along with an addition from the *West Midlands* region's eastern counties, form the smaller of the found clusters. Its northern half follows more or less the boundaries of the administrative regions, but the formed cluster lost its northern territories to the extreme northern cluster of *England*. The region of *West Midlands* of *England* kept its central part intact, but - in addition to its lost of its eastern counties to the central cluster - it also lost the area of its south-western extreme to the cluster of Wales. The north-western area has two separate clusters. Keeping its eastern frontier almost intact, its southern cluster penetrates to the territories of *Northern Wales* region. Its northern cluster shows a well defined separation line between *England* and *Scotland*, and a somewhat fuzzy border on the east. The north-eastern cluster being the last one from *England* also showcases a well defined frontier on the north, with a few satellite pixels from the *Greater London* and the southern part of the *West Midlands* clusters. As discussed above, the region of *Wales* - in addition of containing a few satellite pixels, mostly from *England* -, has a loss, as a small portion of its territories on the north and central east belong to the western clusters of *England*. On the other hand, this is balanced out by a gain on the southern border. *Northern Ireland* has a few satellite pixels from *Scotland*, *Wales* and *England*, mostly on its shores. *Scotland* has its own cluster and its geographic cohesiveness is only disturbed in its northern areas by various satellite pixels, where the density of Twitter users appears to be the least reliable.

Spain

The southern region of *Andalucia* has one of the highest population levels of the Spanish autonomous communities, a factor that is probably causing it being easily divided into three parts, each formed as a cohesive unit respectively by the three western, the three eastern and the two central provinces. Next, the eastern region of *Valencia* is also clearly partitioned into two parts, the southern province of *Alacant* forming its own cluster, only losing its extreme north area. The large central region of *Castilla-La Mancha*

forms a cluster while losing the majority of the pixels of the province of *Cuenca* to its eastern neighbor cluster and the pixels of its northern province *Guadalajara* to the strong cluster of *Madrid*. The latter forms the strong compact central cluster with a halo expending beyond its administrative borders gaining the neighboring territories from the eastern parts of both of the provinces of *Segovia* and of *Avila* and the northern border of *Toledo* on the south. *Madrid* also is the cluster with the most extensive outreach through satellite pixels scattered evenly in every direction. Other units formed by more than a single administrative region are the cluster of *Catalonia* joined by the *Balearic Islands* located at its close proximity. This cluster also has satellite presence across the country. The autonomous community of *Asturias* forms a compact cluster with its southern neighbor region of the province of *Leon*. The region of the autonomous community of *Basque Country* forms also a strong and extensive cluster, joined by more than one of its neighbors: *Foral de Navara* on the east, *Cantabria* on the west, *Rioja* on the south. It also occupies the northern pixels of the province of *Burgos*, its south-western neighbor. It has a few satellites expending mostly to the western provinces. The region of *Aragon* forms a cluster, occupying most of the province of *Soria* on its west, but loosing its eastern and western borders to its neighbors. The remaining parts of *Castile and Leon* form a cluster.

France

Going to the north, we have the loosely connected west-central cluster joining together *Limousin* and *Poitou-Charentes*. Another cluster is formed covering most of *Picardie* and *Haute-Normandie* and the majority of the pixels forming the region of *Centre*. The two remaining large clusters are the western cluster of *Basse-Normandie*, *Bretagne* and *Pays-de-la-Loire*, and the eastern cluster of *Alsace* and *Lorraine*. Latter has significant presence in the main cities of its neighboring regions.

Germany

In *Neithersachtsen* the main cluster contains the pixels of *Bremen* and the norther part of its souther neighbor as well, but is disturbed by satellites from different communities. To its south, a cluster around the cities of *Bonn*, *Cologne*, *Dusseldorf*, *Dortmund* and *Munster* for the cluster covering the majority of the territories of *Nordrhein-Westfalen* with evenly spread satellites. To its south a cluster is formed around the city of *Frankfurt*, occupying the southern half of the region of *Hessen* and having loosely connected parts in the rather sparsely covered regions of *Reinland-Pfaltz* and *Saarland*. The south-western cluster if mainly formed around *Stuttgart*, but covering the majority of the region of *Baden-Wurtenberg*, having a fuzzy border with its northern neighbors. The cluster occupying the majority of the territories of *Bayern* is also divided into loosely connected urban concentrations, like the center around the cities of *Munich*, *Augsburg* and *Nurnberg*. Continuing on the eastern half to the north we have a scattered cluster spread in the three regions of *Sachsen*, *Sachsen-Anhalt* and of *Thuringen*. A very sparse and far from cohesive cluster is formed by the small urban areas following the northern segment of the country's border.

Asia

Southeast Asia with China and Japan

A single cluster covers effectively the whole of the Chinese pixels and it also includes the majority of the pixels of the small state of *Brunei* situated on the neighboring island of *Borneo*. In reason of the relatively small size of the latter, this inclusion can be an artifact as a result of the above mentioned tendency of the clustering method to melt smaller units into the bigger ones with higher probability. While this is as far as this cluster's outreach goes, we note that the Chinese territory contains at least one satellite pixel from each of the other clusters with a few of them having a more significant presence.

Japan is densely occupied. With insignificant presence of satellites from other communities it forms its own cohesive cluster that is the largest one with more than $3,000$ pixels and with a relatively week

outreach into various parts of *China* and the rest of the northern part of the region. *Thailand* forms the second largest cluster. It is cohesive too, and has mostly outreach to it immediate vicinity and *China* as well.

The next three clusters show comparable sizes. While *Malaysia* forms a cluster with a few satellites evenly spread, the Philippines is covered by a cluster that has a more pronounced outreach. It has significant presence in *Vietnam*, *Cambodia*, *Laos* and *China* with a few additional satellites in the norther part of the region. The third similarly sized cluster is the largest of the several communities covering the territories of *Indonesia*. While this country is very well represented, it is also segmented. Its pixels are divided into several widespread clusters. The largest is remarkably concentrated on the central part of the southern island, over *Yogyakarta* and *Central Java* and also covers the vast majority of the pixels covering its northern island, i.e. the regions of *Kalimanthan*, and evenly spread with strong presence in western and eastern parts of the country. It has only a small outreach to *Vietnam*, *Malaysia* and *China*.

In addition, there are five medium-sized and three small clusters, all of them being smaller than the Chinese one. The west-northern part of the Indonesian territories, i.e. the northern part of the *Sumatra* is the cohesive core of the next cluster that also has evenly spread outreach within *Indonesia*, and only a few satellites outside of the country. The next cluster is overly widespread in *Sumatra*, *Borneo* as well as the eastern part of *Indonesia*, but its core is at the western extreme of the densely populated island of *Java*. It also has week outreach to *China*. The next cluster is rather dense on the island of *Sulawesi*, and has average outreach to the rest of the country. *Indonesia* has an additional cluster comparable in size, but it has the center located in the region of *East Java*. Its outreach is less pronounced, similarly evenly spread inside the country, but also reaches the northern countries of the region. Three smaller regions formed by only a few hundred pixels are respectively centered on the western regions of *Java*, on the southern part of the island of *Sumatra*, and on *Bali*. Their outreach is also significantly smaller.

Japan

The country is cut into ten parts and their boundaries loosely follow the administrative subunits. Most of them form cohesive communities in the geographic space but satellites are heavily present in each of them. *Hokkaido* is rather sparsely covered with active pixels. Its core is denser around the large city of *Sapporo*. The region of *Tohoku* has better representation and is covered by a well defined cluster that extends slightly over its west southern neighbor. The neighboring region of *Kanto* is partitioned into two clusters. The region surrounding *Tokyo* has its own smaller cluster with attached part from the west northern extreme of *Kanto* and visible satellite presence throughout the country. The next region is *Chubu*, and it is similarly covered by two clusters. Its northern coast with the west central part and its southern coast with the west central part are separated. The latter has attached the eastern territory that is part of the next administrative region of *Kinki*. The rest of the latter forms its cohesive unit with visible satellite presence mostly in the closer regions. The regions of *Shikoku* and *Chugoku* form a single cluster, but the western part of the latter is attached to the last cluster that is covering also the region of *Kyushu* on the west of the country. While the northern part of *Okinava* is also attached to it, the rest of the small islands are covered by various satellites and a very small southern cluster at the extreme.

India

The cluster covering most of the active pixels of *Tamil Nadu* is situated on the south east of the country. It has several denser pots around cities, the biggest one being around *Chennal* on the north extreme of the eastern coast of the state. Only a few satellites reach out to the north of the country. The cluster covering the majority of the pixels of the state of *Karnataka* is only dense around the cities of *Bangalore* and *Mangalore*. The rest of the state is very sparsely occupied by active geo-users, but its cluster has a weak satellite presence evenly spread in the rest of the country. The south western state of *Kerala* is rather well represented and forms a very cohesive community with weak outreach. The next cluster

is still medium-sized but is very diffuse. Its only cohesive core is centered around the city of *Mumbai* on the west coast of the state of *Maharashtra* that is otherwise very weakly represented. This cluster has satellites evenly spread. The next community is occupying the majority of the pixels of the state of *Gujarat* with representation mainly around the larger cities and an evenly spread but weak satellite presence. The remaining two smallest clusters are formed also by geographically not cohesive urban areas that are nonetheless in neighboring areas of the country. The first is the cluster formed by the denser core around the metropolitan area of *Prune* and the other core formed by the pixels of *Panajim*, the capital of the state of *Goa*. The smallest cluster occupies most of the pixels of the state of *Andhra Pradesh* having its core formed around its capital, *Hyderabad* having even but weak outreach and most of the pixels in the east northern state of *Assam*.

5 Description of the SI data sets

We provide as additional SI the S2 File. It contains a series of regional graph data sets with the necessary information to reproduce the presented community projection results.

- `<region>.dat`: the graph community information file, multicolumn text format starting with a header line. The node is identified in the first column by the healpix_id followed by the cluster_id of the pixel. Where there are partial second-level clustering results they are combined with the rest of the results (as in the corresponding figures) and an additional column shows the L2_cluster_ids, with the unchanged nodes remaining in their original community, but all clusters renumbered from 1. center_lon and center_lat indicate the pixel center's location while the polygon column gives the HEALPix in a Well Known Text (WKT) format with eight-point precision.
- `<region>-hist.dat` files: the histogram data file necessary for the reproduction of the user number distribution Figures C-E in S1 File. A simple tab separated 2-column file without header line.

References

1. Newman ME, Girvan M. Finding and evaluating community structure in networks. Phys Rev E. 2004;69(2):026113.
2. Brandes U, Delling D, Gaertler M, Görke R, Hoefer M, Nikoloski Z, et al. On finding graph clusterings with maximum modularity. In: Graph-theoretic concepts in computer science. Springer International Publishing; 2007. p. 121–132.
3. Newman MEJ. Fast algorithm for detecting community structure in networks. Phys Rev E. 2004;69(6):066133.
4. Ovelgönne M, Geyer-Schulz A, Stein M. Randomized greedy modularity optimization for group detection in huge social networks. In: Proc. SNA-KDD 10; 2010. .
5. Geyer-Schulz A, Ovelgönne M. The Randomized Greedy Modularity Clustering Algorithm and the Core Groups Graph Clustering Scheme. In: German-Japanese Interchange of Data Analysis Results. Springer International Publishing; 2014. p. 17–36.
6. Wagner S, Wagner D. Comparing Clusterings - An Overview; 2007.
7. Rand W. Objective criteria for the evaluation of clustering methods. Journal of the American Statistical association. 1971;66(336):846–850.
8. Hubert L, Arabie P. Comparing partitions. Journal of Classification. 1985;2(1):193–218.
9. Strehl A, Joydeep G. Cluster ensembles - a knowledge reuse framework for combining multiple partitions. The Journal of Machine Learning Research. 2003;3:583–617.
10. Challenging the access ban in Turkey;. Accessed 1 September 2014. https://blog.twitter.com.
11. Turkey Twitter ban: Constitutional court rules illegal;. Accessed 2 April 2014. http://www.bbc.com/news.

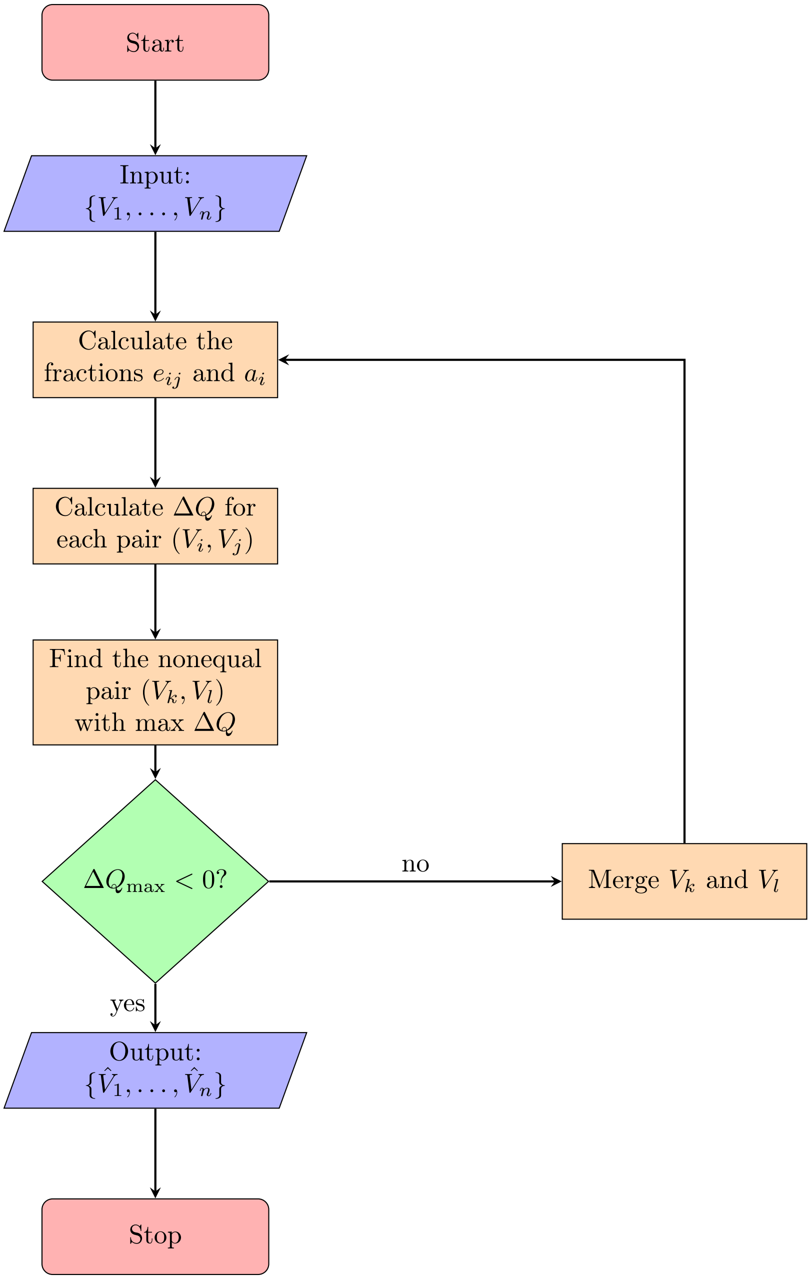

Figure A. Flowchart of simple greedy modularity clustering algorithm

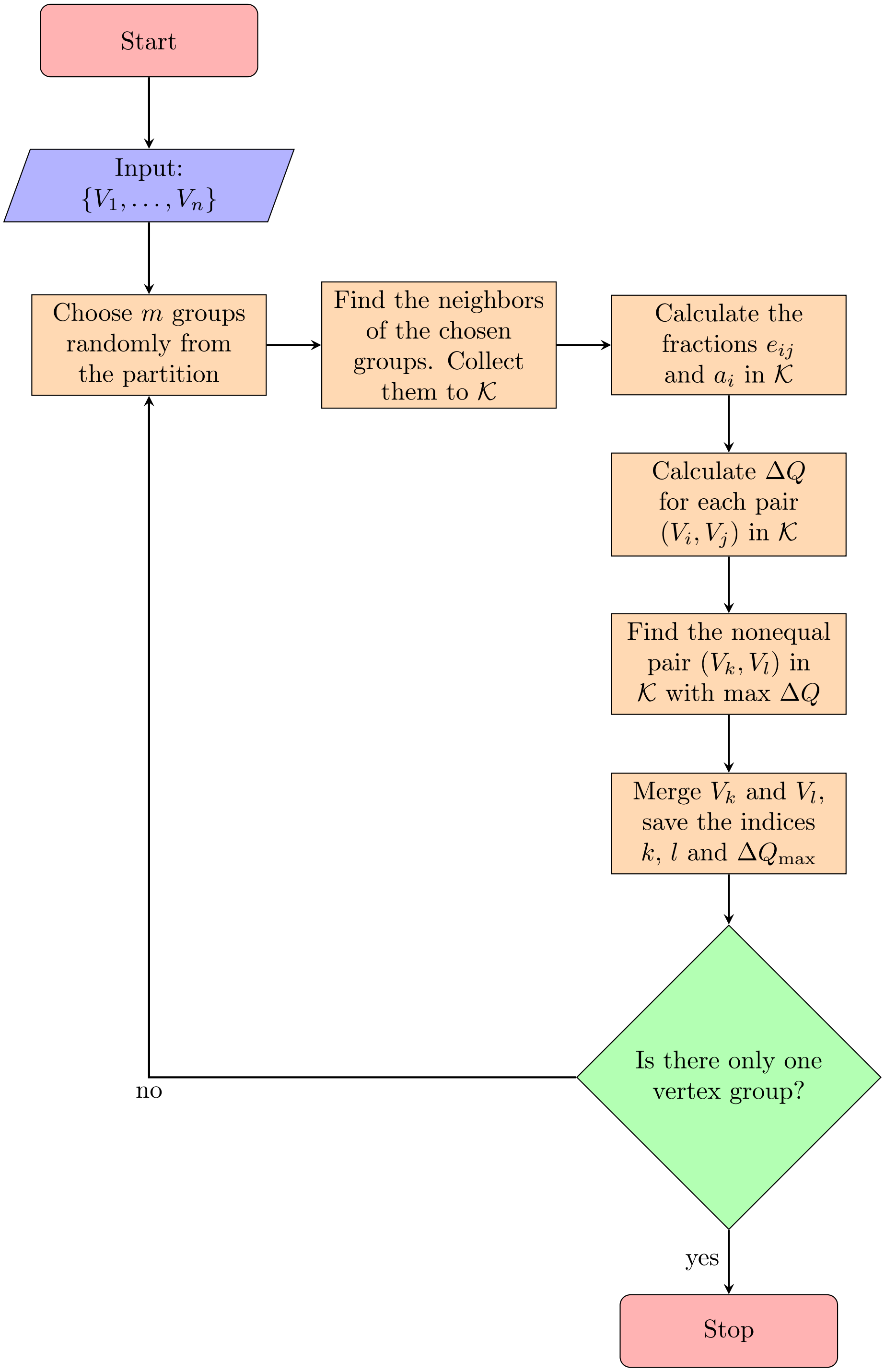

Figure B. Flowchart of randomized greedy modularity clustering algorithm

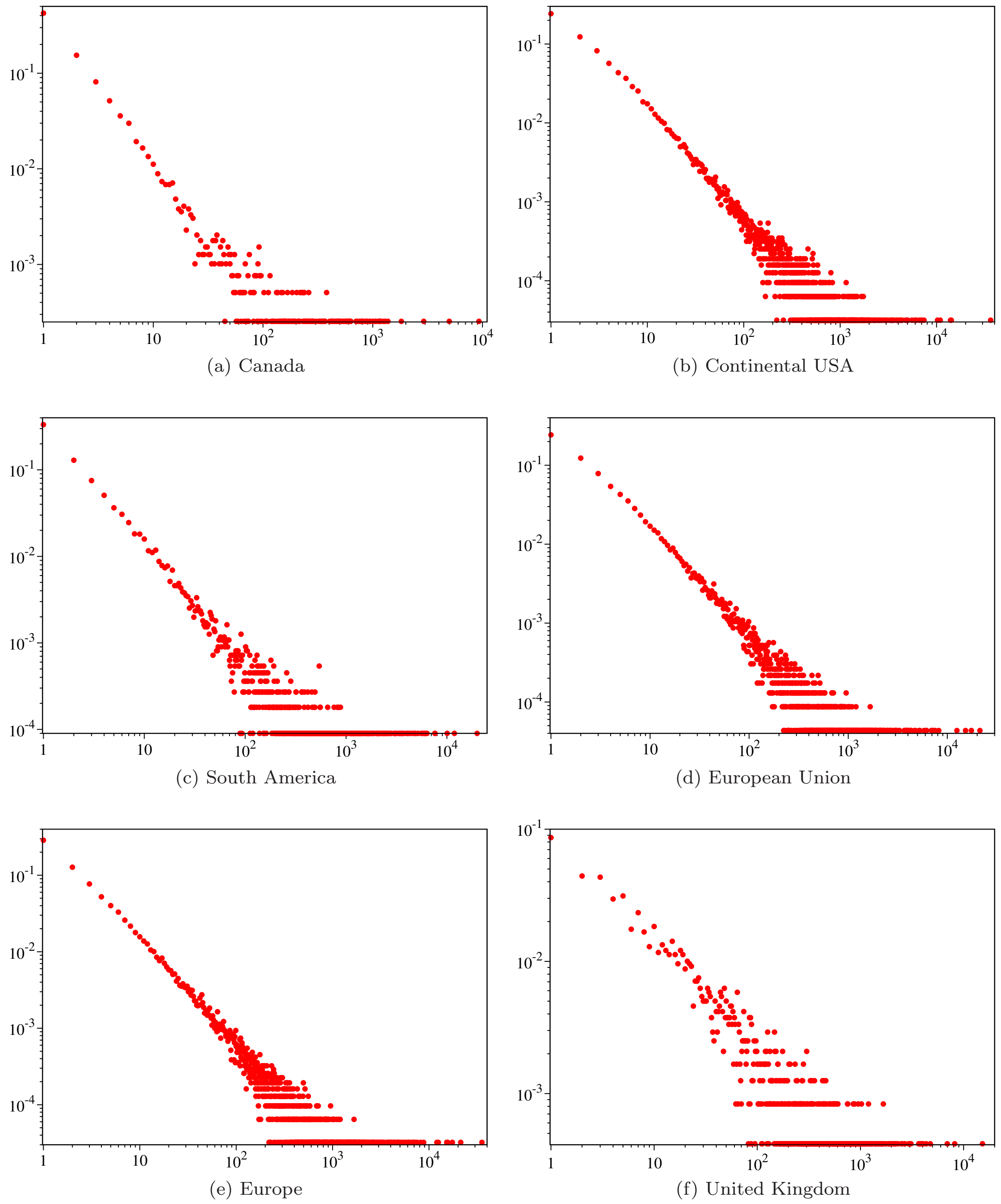

Figure C. Distribution of number of users in a pixel of a region under consideration. Part I

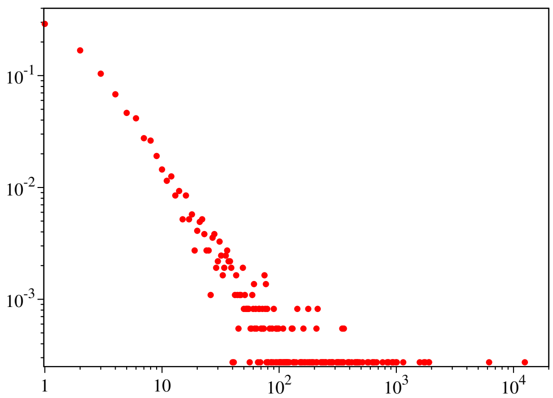

(a) France

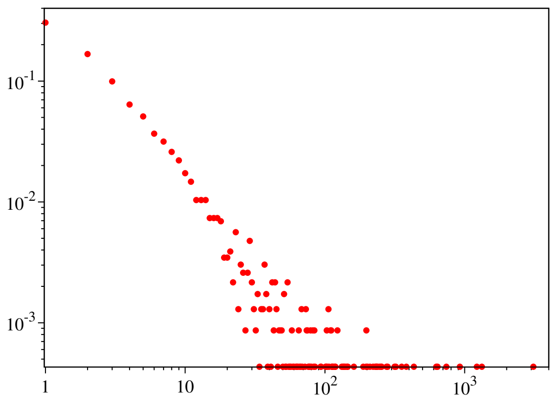

(b) Germany

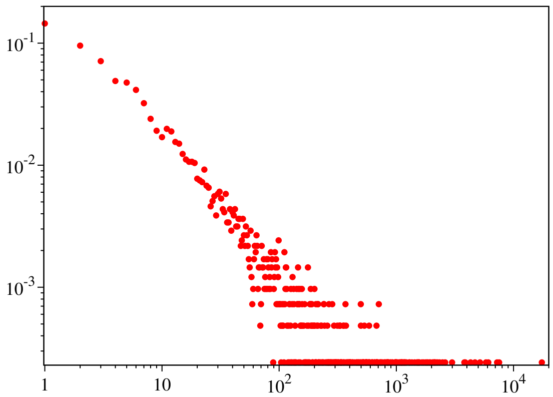

(c) Spain

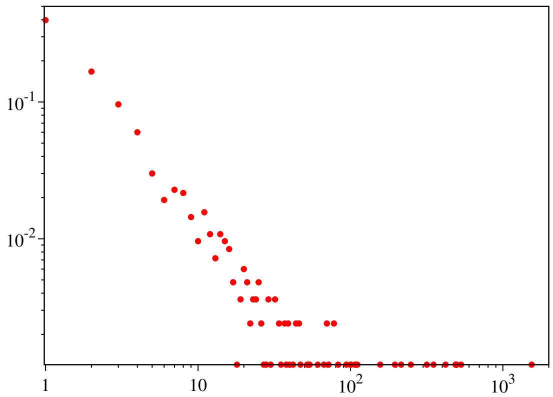

(d) Former Yugoslavia

Figure D. Distribution of number of users in a pixel of a region under consideration. Part II

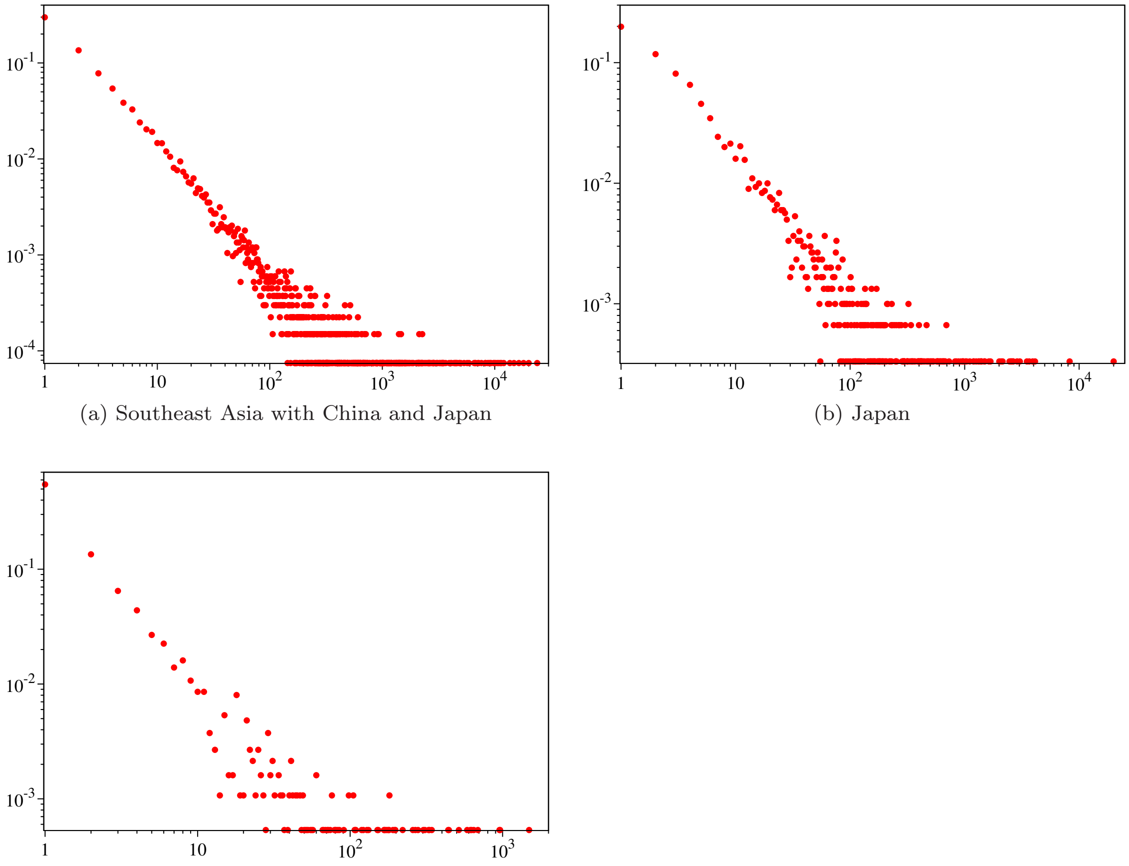

(a) Southeast Asia with China and Japan

(b) Japan

(c) India

Figure E. Distribution of number of users in a pixel of a region under consideration. Part III
